# Chemical Synthesis of Site-Specifically 2′-Azido-Modified RNA and Potential Applications for Bioconjugation and RNA Interference

**DOI:** 10.1002/cbic.201000646

**Published:** 2010-12-17

**Authors:** Michaela Aigner, Markus Hartl, Katja Fauster, Jessica Steger, Klaus Bister, Ronald Micura

**Affiliations:** [a] Institute of Organic Chemistry, Center for Molecular Biosciences CMBI, University of Innsbruck6020 Innsbruck (Austria), Fax: (+43) 507-512-2892; [b] Institute of Biochemistry, Center for Molecular Biosciences CMBI, University of Innsbruck6020 Innsbruck (Austria)

**Keywords:** azides, gene silencing, labeling, phosphoramidite, siRNA, solid-phase synthesis

One of the most important scientific discoveries in the recent past concerns RNA interference (RNAi), which is a post-transcriptional gene-silencing mechanism induced by small interfering RNA (siRNA) and micro-RNA (miRNA).[1] RNAi has opened up new avenues in the development of siRNA and miRNA as therapeutic agents for various diseases.[2] The reason for the large number of reports about chemically modified siRNA is their potential to enhance nuclease resistance, to prevent immune activation, to decrease off-target effects, and to improve pharmacokinetic and pharmacodynamic properties, all of which are important for the application of siRNA as therapeutic agents.[3] Another substantial challenge is siRNA delivery, because these reagents cannot easily traverse cell membranes because of their size and negative charge.[4] To date, the most promising therapeutic approach based on RNAi involves chemically modified siRNA that can resolve some of the issues mentioned above.

Chemical siRNA modifications belong to four classes—backbone, ribose, nucleobase, and terminal modifications—with ribose modifications being the most common.[2b] Structurally simple alterations, such as 2′-OCH_3_ and 2′-F, lead to significantly enhanced performance of siRNA with diverse target genes, provided that they are positioned in a site-specific manner.[3] In particular, 2′-F modifications possess the extraordinary property of being very well accepted onto the guide (antisense) strand, while most other modifications are much better tolerated by the passenger (sense) strand.[5] The guide strand is incorporated into the crucial functional particle, the RNA-induced silencing complex (RISC); thus RNA recognition and discrimination from non-native counterparts is very stringent.[6]

**Scheme 1:**
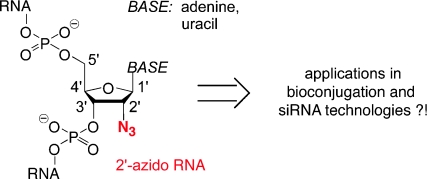
Solid-phase chemical synthesis of RNA with site-specifically 2′-azido-modified nucleosides, and the potential of these novel RNA derivates for bioconjugation reactions and siRNA technologies.

We postulated that siRNA with specific 2′-azido groups (2′-N_3_) should have the potential for enhanced performance, because this functional group is small, polar, and supports the C3′-endo ribose pucker[7] that is characteristic for an A-form RNA double helix. Interestingly, this modification has not yet been explored for siRNA technologies. It is even more surprising that, to the best of our knowledge, the solid-phase chemical synthesis of 2′-azido-modified RNA has not yet been described.[8] The prospect of potential siRNA applications, and also of promising applications in modern bioconjugation chemistry (such as Staudinger ligation and click chemistry)[9] prompted us to take up the challenge of synthesizing these RNA derivatives (Scheme 1).

Nucleosides that carry an azido group—no matter at which position—cannot be isolated in the form of stable phosphoramidite building blocks, the most convenient form for use in advanced RNA solid-phase synthesis approaches.[10] This is due to the inherent reactivity between phosphor-III species and azides according to the Staudinger reaction. However, we had experimental indications that this reactivity becomes prevalent only at very high concentrations of azido-modified nucleoside phosphoramidites (e.g., upon evaporation of the solvents to isolate these compounds). This led us to consider the possibility that strand assembly by phosphoramidite chemistry might still be feasible (at reasonable phosphoramidite concentrations) once the azide moiety has been incorporated into the RNA. Additionally, two very recent independent studies, one on the chemical synthesis of 4′-azidomethyl-thymidine modified DNA and the other on the circularization of DNA by using a solid-support bearing an azido linker, encouraged us to target 2′-azido-RNA synthesis.[11a,b]

We decided to focus on the 2′-azido-2′-deoxyuridine and the 2′-azido-2′-deoxyadenosine building blocks as 2-chlorophenyl phosphodiester derivatives, **1** and **2**, with the aim of employing them later in a single cycle of standard RNA phosphotriester coupling for the incorporation into RNA, while strand assembly should follow standard phosphoramidite chemistry.

**Scheme 2:**
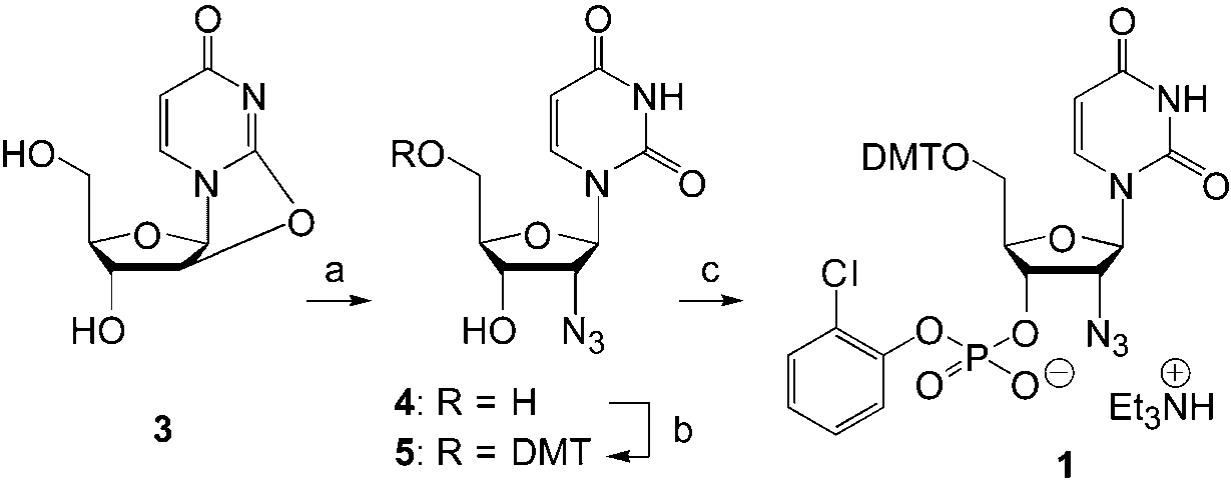
Synthesis of the 2′-azido-2′-deoxyuridine 3′-phosphodiester building block **1** for RNA solid-phase synthesis. Reagents and conditions: a) 1.8 equiv LiF, 1.8 equiv (CH_3_)_3_SiN_3_, in *N*,*N*,*N′*,*N′*-tetramethylethylenediamine (TMEDA)/DMF 1:1, 110 °C, 48 h, 66 %; b) 1.5 equiv 4,4′-dimethoxytriphenylmethyl chloride (DMT-Cl), in pyridine, RT, 24 h, 69 %; c) i: 4 equiv *N*-methylimidazole, in THF, RT, 5 min; ii: 2.5 equiv 2-chlorophenyl phosphorodichloridate, 5.5 equiv 1,2,4-triazole, 5 equiv Et_3_N, in THF, RT, 10 min, 86 %.

For building block **1**, we started the synthesis with 2,2′-anhydrouridine **3** (Scheme 2) which is commercially available or can be obtained from uridine by a single transformation by using diphenyl carbonate in DMF.[12] Nucleophilic ring-opening with in situ-generated lithium azide in DMF furnished the 2′-azido-2′-deoxyuridine derivative **4**.[13] Subsequently, the 5′-hydroxyl group was protected as dimethoxytrityl (DMT) ether to form compound **5**.[14] Conversion into the corresponding phosphodiester **1** was achieved in good yield by reaction with in situ-generated 2-chlorophenyl chlorophosphorotriazolide, analogously to a general procedure in the literature.[15]

**Scheme 3:**
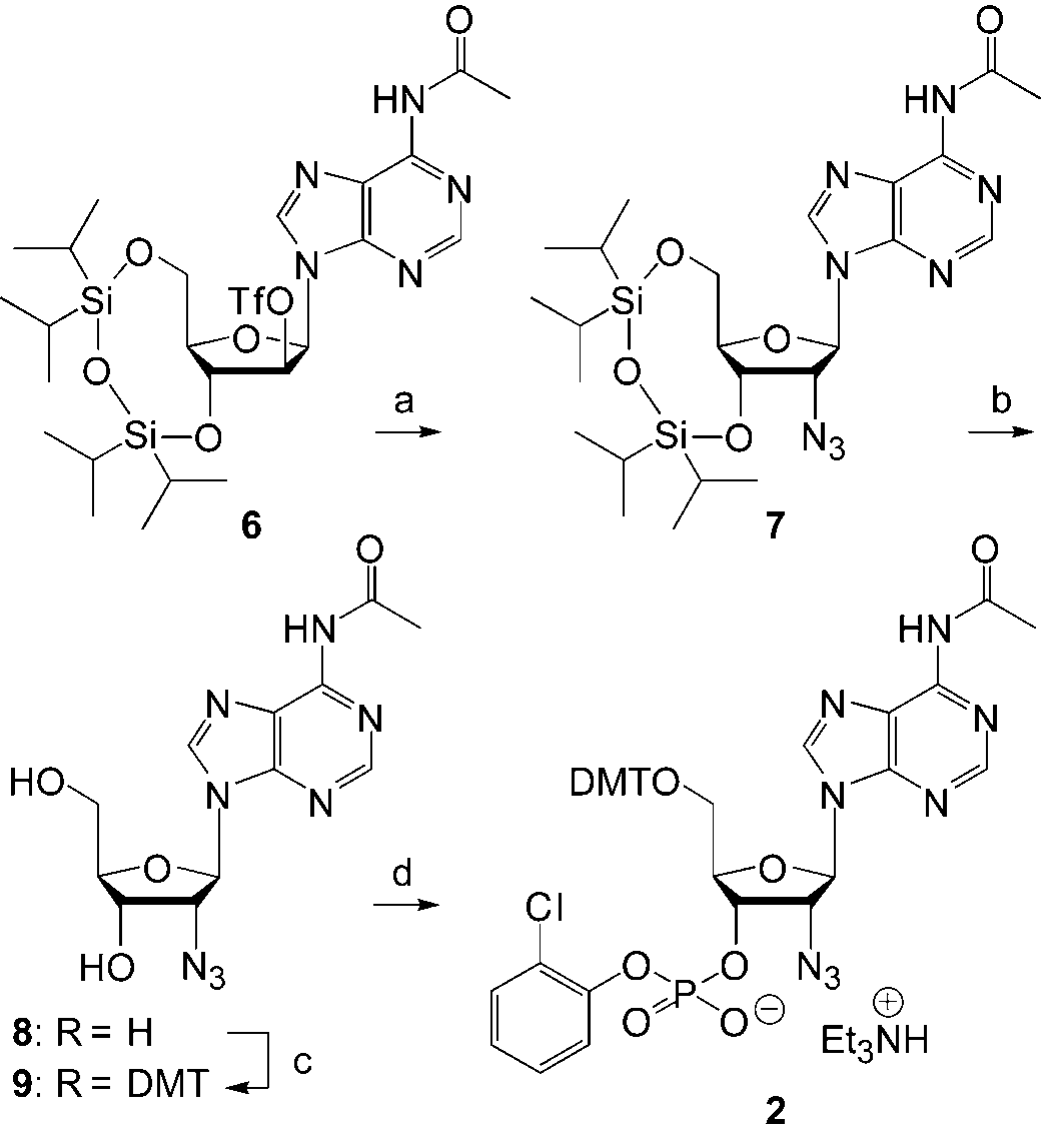
Synthesis of the 2′-azido-2′-deoxyadenosine 3′-phosphodiester building block **2** for RNA solid-phase synthesis. Reagents and conditions: a) 5 equiv LiN_3_, in DMF, RT, 20 h, 86 %; b) 1 m TBAF, 0.5 m CH_3_COOH, in THF, RT, 2 h, 71 %; c) 1.5 equiv DMT-Cl, in pyridine, RT, 16 h, 64 %; d) i: 4 equiv *N*-methylimidazole, in THF, RT, 5 min; ii: 2.5 equiv 2-chlorophenyl phosphorodichloridate, 5.5 equiv 1,2,4-triazole, 5 equiv Et_3_N, in THF, RT, 10 min, 79 %.

For building block **2**, we started the synthesis with the triflated adenosine derivative **6**, which was readily obtained from adenosine according to the previously described synthesis to 2′-methylseleno adenosine (Scheme 3).[16] Treatment of **6** with lithium azide in DMF furnished key compound **7**, followed by cleavage of the silyl protecting group to give the diol derivative **8**. The 5′-hydroxyl group was then protected as the DMT ether to form **9**. Conversion into the corresponding phosphodiester **2** was achieved in good yield by treatment with 2-chlorophenyl chlorophosphorotriazolide.

**Figure 1:**
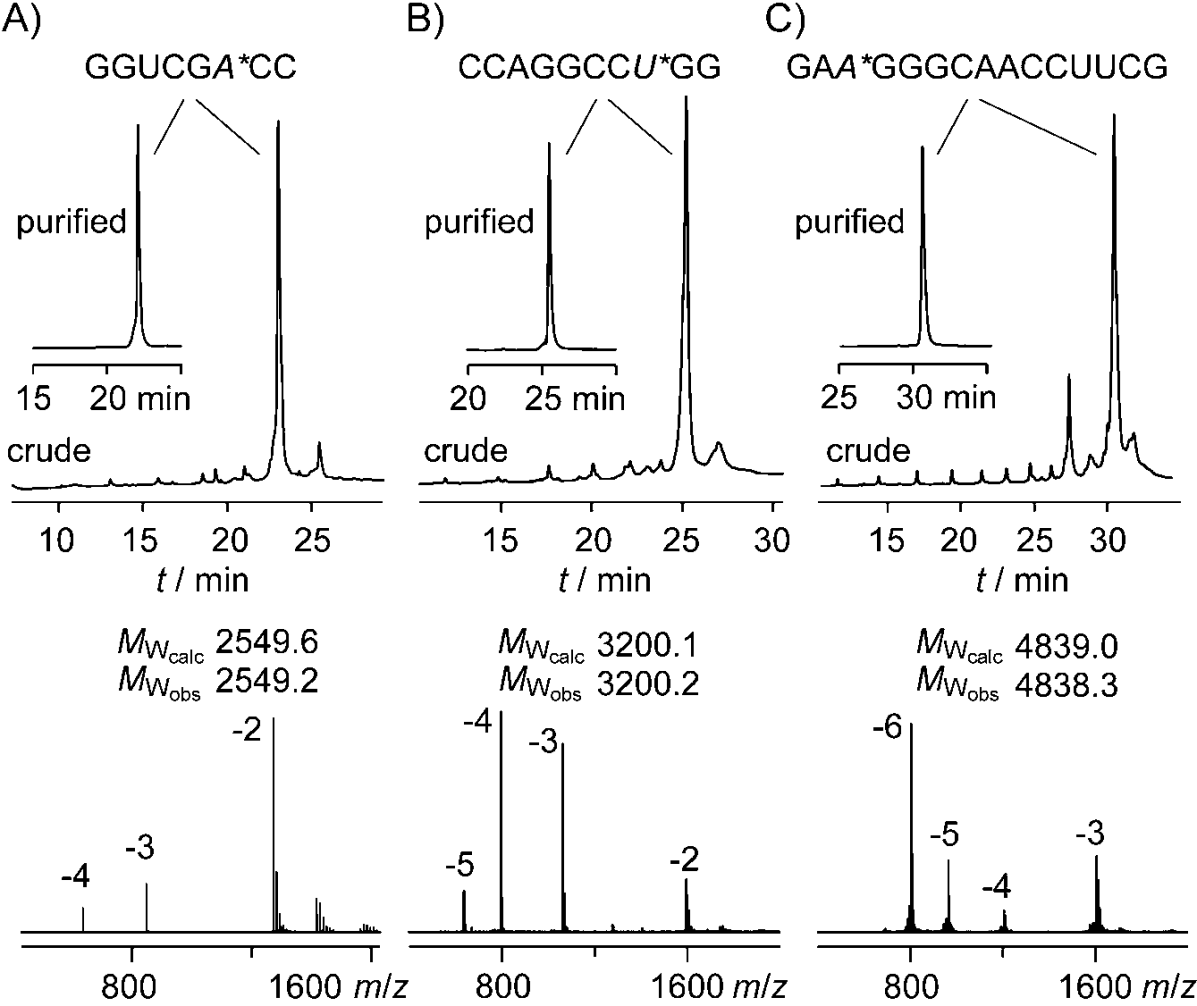
Characterization of 2′-azido-modified oligoribonucleotides by anion-exchange HPLC and LC-ESI mass spectrometry. A) 8 nucleotide (nt) RNA containing 2′-azido-2′-deoxyadenosine. B) 10 nt RNA containing 2′-azido-2′-deoxyuridine. C) 15 nt RNA containing 2′-azido-2′-deoxyadenosine. For conditions see the Supporting Information.

For the incorporation of the 2′-azido-modified nucleoside phosphodiester building blocks **1** and **2** into RNA, we conducted strand assembly by automated standard RNA solid-phase synthesis with 2′-*O*-[(Triisopropylsilyl)oxy]methyl (2′-*O*-TOM)-protected nucleoside phosphoramidites[17] up to the position of the intended azide modification. The synthesis was interrupted after the detritylation step that liberated the terminal 5′-hydroxyl group. Coupling of the phosphodiester building block, **1** or **2,** was achieved by activation with 1-(mesitylene-2-sulfonyl)-3-nitro-1,2,4-triazole (MSNT) by using two syringes attached to the column for reagent delivery and mixing under argon.[18] By using this simple setup (see the Supporting Information), coupling yields higher than 95 % were achieved. After manual capping, strand elongation was continued by standard automated RNA phosphoramidite chemistry. With sequences of up to about 25 nucleotides, we had no indication (from inspection of oligonucleotide by-products by LC-ESI mass spectrometry; see also refs. [10b] and [11a, c]) that the ribose 2′-azido group had reacted with the phosphoramidite moiety under the coupling conditions. For deprotection, the oligoribonucleotides containing 2′-azido groups were first treated with *syn*-2-pyridine aldoxime/tetramethylguanidine in dioxane/water to cleave the 2-chlorophenyl phosphate protecting groups.[18] Then, standard deprotection conditions were applied by using CH_3_NH_2_ in ethanol/water followed by treatment with 1 m tetra-*n*-butylammonium fluoride (TBAF) in THF. After purification by anion-exchange HPLC, the expected molecular weights were confirmed by liquid chromatography ESI (LC-ESI) mass spectrometry (Table 1, Figure 1).

**Table 1:** Selection of synthesized 2′-azido-2′-deoxyuridine (U*) and 2′-azido-2′-deoxyadenosine (A*) containing oligoribonucleotides

Sequence (5′→3′)	Amount [nmol]	*M*_W calcd_ [amu]	*M*_W obs_ [amu]
GGU*CGACC	125	2549.6	2549.2
GGUCGA*CC	196	2549.6	2549.2
CCAGGCCU*GG	87	3200.1	3200.2
CCA*GGCCUGG	79	3200.1	3200.2
GAAGGGCAACCU*UCG	173	4839.0	4838.4
GAA*GGGCAACCUUCG	161	4839.0	4838.3
G_2_UCUCU*GCCA_2_UA_2_GACATT	298	6678.1	6677.8
UGUCU_2_A*U_2_G_2_CAGAGAC_2_TdG	120	6697.1	6696.8
UGUCU*UAUU*G_2_CAGAGAC_2_TdG	55	6722.1	6721.5
UGUCU_2_AUU*G_2_CA*GAGAC_2_TdG	68	6722.1	6721.2

**Figure 2:**
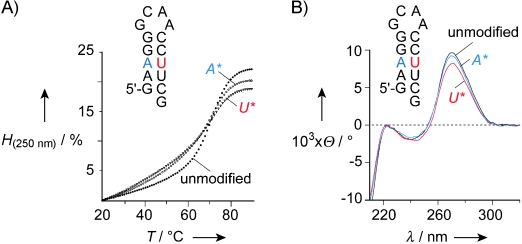
Spectroscopic characterization of 2′-azido-modified oligoribonucleotides. A) Overlay of UV melting profiles of GAAGGGCAACCUUCG, GAAGGGCAACCU*UCG, GAA*GGGCAACCUUCG, *c*_RNA_=5 μm; B) Overlay of CD spectra of GAAGGGCAACCUUCG, GAAGGGCAACCU*UCG, GAA*GGGCAACCUUCG, *c*_RNA_=2 μm. Conditions: 10 mm Na_2_HPO_4_, 150 mm NaCl, pH 7.0. U*=2′-azido-2′-deoxyuridine, A*=2′-azido-2′-deoxyadenosine, *H*=hyperchromicity.

Next we investigated the influence of the 2′-azido group on the thermal stability of RNA double helices by using temperature-dependent UV spectroscopy (Figure 2A). At 150 mm NaCl and 10 mm Na_2_HPO_4_ (pH 7.0), the unmodified hairpin 5′-GAAGGGCAACCUUCG melted at 72.5±0.5 °C while the 2′-azido-modified counterparts, 5′-GAAGGGCAACCU*UCG and 5′-GAA*GGGCAACCUUCG, displayed *T*_m_ values of 71.7±0.5 °C and 71.6±0.5 °C, respectively. This demonstrates that the 2′-azido group is modestly destabilizing, which might arise from a slight steric interference of the moiety with the 3′-phosphate.[7a] Additionally, we examined this hairpin system with CD spectroscopy: the spectra clearly indicate that the overall conformation of a typical A-form double helical geometry is retained in the 2′-azido-modified RNA (Figure 2B).

**Figure 3:**
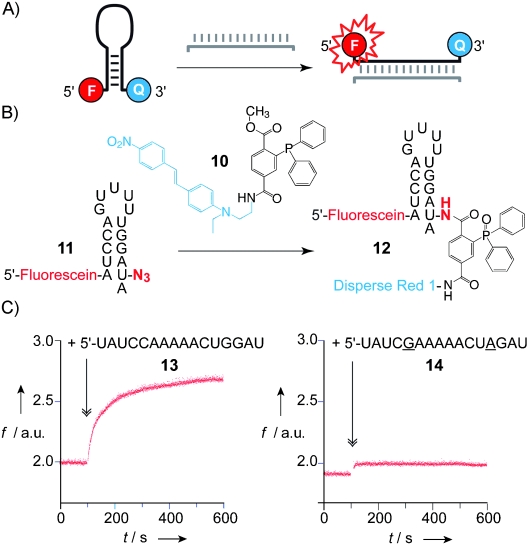
Attachment of a fluorescence quencher to RNA with an internal site-specific ribose 2′-azido group. A) Concept of molecular beacon (MB); B) Staudinger ligation of the phosphine derivative **10** to RNA **11**; C) Timedependent fluorescence response of the MB **12** to addition of the target sequence **13** (left) and of the near-cognate sequence **14** (right) with double mismatch discrimination (mismatched nucleobases underlined). Conditions: *c*_MB_=0.5 μm, *c*_target_=2.5 μm, 100 mm MES⋅NaOH, 1.0 mm MgCl_2_, pH 6.5. *λ*_ex_=490 nm, *λ*_em_=514 nm. *f*=fluorescence (emission), *t*=time.

To assess the potential of site-specifically 2′-azido-modified RNA for bioconjugation reactions[9] we attached a fluorescence quencher to the azido group to construct a molecular beacon (MB).[19] MBs are widely used probes for the specific detection of DNA and RNA targets, and their function is based on the interaction between a fluorescent (F) and a quenching (Q) moiety (Figure 3A). In the absence of the target, the MB adopts a hairpin structure that brings F and Q into close proximity resulting in quenching of fluorescence. In contrast, hybridization with the target of the MB leads to separation of F and Q and thus fluorescence emission. Here we wished to demonstrate that the attachment of a dye to the 2′-azido position can be performed post-synthetically by following one of the most widespread bioconjugation strategies, the Staudinger ligation.[9a, 20] We synthesized the Disperse Red 1 (DR1) phosphine derivative **10** (see Supporting Information) and then applied typical Staudinger conditions[20] in H_2_O/DMF for ligation to the 5′-fluorescein-labeled RNA **11** (Figure 3B). With a 100-fold excess of **10**, we observed complete conversion of **11** into the amide- (**12**) and the *O*-methyl imidate (**12 a**) linked DR1-RNA conjugates (85 %), and a minor amount (15 %) of the reduced (nonconjugated) 2′-NH_2_ RNA derivative of **11** (see the Supporting Information).

This clearly shows that, despite the relatively high steric hindrance of the ribose 2′-azido group in RNA, its reactivity is still sufficient for efficient chemical ligation. Moreover, the fluorescence responses of **12** depicted in Figure 3C demonstrate the expected functionality for target recognition and for discrimination from near-cognate sequences.

**Figure 4:**
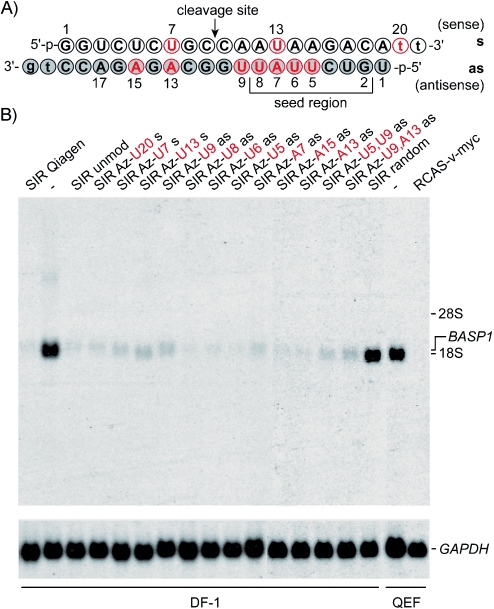
Gene silencing by 2′-azido-2′-deoxyuridine- and 2′-azido-2′-deoxyadenosine-modified siRNA. A) Sequence of the *BASP1* siRNA used in this study;[21a] nucleosides in red indicate positions of 2′-azido modification. B) Biological activities of 2′-azido siRNAs directed against *BASP1* mRNA. Chicken DF-1 cells grown on 60 mm dishes were transiently nucleofected with 0.12 nmol (∼1.5 μg) aliquots of unmodified commercial (Qiagen) or own (unmod) synthetic siRNAs (SIR) directed against the chicken *BASP1* mRNA, or of siRNAs containing azido (Az) groups at the indicated nucleotides on sense (s) or antisense (as) strands. An equal aliquot of siRNA with a shuffled (random) nucleotide sequence was used as a control. Total RNAs were isolated two days after siRNA delivery and 5 μg-aliquots were analyzed by Northern hybridization by using DNA probes specific for the chicken *BASP1* gene, or the quail housekeeping *GAPDH* gene. RNAs (5 μg) from normal quail embryo fibroblasts (QEF), and from QEF transfected with the retroviral construct RCAS-v-myc[21b] encoding the *BASP1*-suppressing v-Myc oncoprotein were used as controls. The electrophoretic positions of ribosomal RNA are indicated in the margin. All siRNAs depicted contain overhangs of 2′-deoxynucleosides (lower case letters). SIR random: 5′-UCUGGGUCUAAGCCAAACAUT/5′-UGUUUGGCUUAGACCCAGAUdG.

In order to evaluate the potential of 2′-azido-modified oligoribonucleotides in siRNA applications, we employed a model system for the knockdown of the brain acid soluble protein 1 (BASP1) gene by transient siRNA nucleofection in the chicken DF-1 cell line by using standard commercial siRNA duplexes as previously described.[21a] Expression of the *BASP1* gene is specifically suppressed by the Myc oncoprotein, while the BASP1 protein is an efficient inhibitor of Myc-induced cell transformation.[21a] We synthesized ten siRNA duplexes for the Myc target *BASP1* with a single 2′-azido-2′-deoxyuridine or 2′-azido-2′-deoxyadenosine modification and the sequence organisation depicted in Figure 4A (see also Supporting Information). All the modified siRNAs caused gene silencing to an extent that was comparable to that of the unmodified reference duplex (Figure 4B). Remarkably, the 2′-azido group is very well tolerated in the guide strand, even when the site of modification is located in the seed region or very close to the actual cleavage site. Moreover, double azido-labeled siRNAs, one embracing the site of cleavage (SIR Az-U9A13) and another with the modifications at the seed region (SIR Az-U5, U9), also showed efficient silencing activity (Figure 4B). These experiments demonstrate that the 2′-azido group adds to the limited selection of modifications (2′-F and 2′-OCH_3_) that are excellently tolerated in the guide strand.[3d, 5, 6a, 22]

In conclusion, the solid-phase synthesis approach presented here efficiently yields RNA with site-specific 2′-azido groups, and provides a reliable foundation for a wide range of applications in modern bioconjugation strategies and siRNA technologies. Clearly, to evaluate the full potential of this type of modification, systematic studies of 2′-azido-modified siRNA on nuclease resistance, immune activation, off-target effects, toxicity, membrane permeability, and delivery will be required. We are confident that, in particular, the small size and the high polarity of the azido group will provide improvements for at least some of these effects. Moreover, the 2′-azido group in siRNA is available for the attachment of hydrophobic tags (such as cholesterols, lipids, cofactors, cell-penetrating peptides) to enhance delivery properties. Additionally, the attachment of fluorescent dyes to the azido anchor (as exemplified here in the context of MBs) facilitates the localization and tracking of siRNA in the cell. Another feature that has to be explored in future studies of 2′-azido-RNA (and in particular of 2′-azido-siRNA) is photoreactivity, and whether it can be exploited for crosslinking to peptide and protein targets. All these aspects represent the focus of ongoing studies in our laboratory together with the expansion of the concept towards 2′-azido-2′-deoxycytidine- and 2′-azido-2′-deoxyguanosine-containing RNA. At the current state of research, the 2′-azido modification might prove superior to the more than 35 other types of chemical modifications that have been tested for their effects on siRNA performance.[2] It might also prove useful in the manipulation of other types of noncoding RNA in the cell.[23]
