# Supplementary material for: Chemical Synthesis of Site-Specifically 2′-Azido-Modified RNA and Potential Applications for Bioconjugation and RNA Interference
Source: Chembiochem. 2010 Dec 17;12(1):47–51. doi: 10.1002/cbic.201000646 (PMC3193913; doi:10.1002/cbic.201000646)
Supplement: Supplementary file 1 [file cbic0012-0047-SD1.pdf]

CHEMBIOCHEM

Supporting Information

© Copyright Wiley-VCH Verlag GmbH & Co. KGaA, 69451 Weinheim, 2010

Chemical Synthesis of Site-Specifically 2′-Azido-Modified RNA and Potential Applications for Bioconjugation and RNA Interference

Michaela Aigner,[a] Markus Hartl,[b] Katja Fauster,[a] Jessica Steger,[a] Klaus Bister,[b] and Ronald Micura*[a]

cbic_201000646_sm_miscellaneous_information.pdf

CONTENTS

Supporting Methods

1. Synthesis of 2'-azido-2'-deoxyuridine building block **1** S2
2. Synthesis of 2'-azido-2'-deoxyadenosine building block **2** S6
3. Solid-phase synthesis, deprotection and purification of 2'-azido-modified RNA S13
4. Spectroscopic characterization of 2'-azido modified oligoribonucleotides S16
5. Staudinger ligation of 2'-azido-modified RNA for a molecular beacon application S17
6. Table of synthesized 2'-azido-modified RNA sequences S23
7. Table of 2'-azido-modified siRNA duplexes S24
8. Thermal denaturation study of 2'-azido-modified siRNA duplexes S25
9. RNA interference and Northern analysis S26
10. References S27

Supporting Methods

General. ^{1}H, ^{13}C and ^{31}P NMR spectra were recorded on a Bruker DRX 300 MHz instrument. The chemical shifts are reported relative to TMS and referenced to the residual proton signal of the deuterated solvent: $CDCl_3$ (7.26 ppm), [D_6]DMSO (2.49 ppm) for ^{1}H NMR spectra. Referencing for ^{13}C NMR spectra: $CDCl_3$ (77.1 ppm), [D_6]DMSO (39.5 ppm). ^{31}P shifts are relative to external 85% phosphoric acid. ^{1}H and ^{13}C assignments were generally based on COSY and HSQC experiments. MS experiments were performed on a Finnigan LCQ Advantage MAX ion trap instrumentation, samples were analyzed in the positive- or negative-ion mode. Reaction control was performed via analytical thin-layer chromatography (TLC, Macherey-Nagel) on silica plates with fluorescent indicator. Flash column chromatography was carried out on silica gel 60 (70-230 mesh). 5'-Fluoresceinphosphoramidite (6-FAM) was purchased from Metkinen Chemistry. 2'-*O*-TOM standard RNA nucleoside phosphoramidite building blocks were purchased from Glen Research and ChemGenes and the corresponding polystyrene-supports from GE Healthcare (Custom Primer Support, 80 µmol/g; *PS 200*). Chemical reagents and solvents were purchased from commercial suppliers (Sigma-Aldrich, Acros) and used without further purification. Organic solvents for reactions were dried overnight over freshly activated molecular sieves (4 Å). All reactions were carried out under argon atmosphere.

1. Synthesis of 2'-azido-2'-deoxyuridine building block (1)

2'-Azido-2'-deoxyuridine (4)

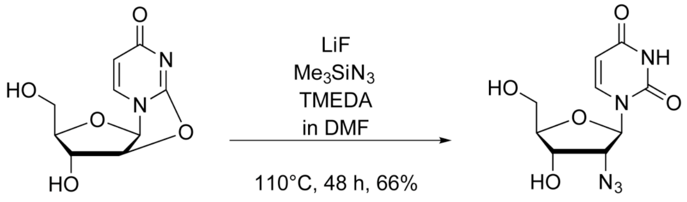

3

$C_9H_{10}N_2O_5$
[226.19]

4

$C_9H_{11}N_5O_5$
[269.21]

Compound **4** was prepared with slight modifications in analogy to the procedure described in reference 1: A suspension of lithium fluoride (115 mg, 4.42 mmol) in dry DMF (4.9 mL) was heated to 105°C followed by the addition of *N*,*N*,*N'*,*N'*-tetramethylethylenediamine (4.9 mL) and trimethylsilylazide (582 µL, 4.42 mmol). After 30 min, 2,2'-anhydrouridine **3** (556 mg, 2.46 mmol) was added and the mixture was kept at 110°C for 48 h. Volatiles were evaporated and the residue was coevaporated twice with methanol. The dark-brown residue was dissolved in methanol (2.46 mL) and EtOAc (9.84 mL) was added for the precipitation of salts and unconverted starting material. After filtration and evaporation of the solvents the crude product was purified by column chromatography on SiO_2 (EtOAc/MeOH, 9/1 v/v). Yield: 436 mg of **4** as yellow foam (66%). ^1H NMR (300 MHz, DMSO-d_6): δ 3.52-3.59 (m, 1H, H(a)-C(5')); 3.61-3.68 (m, 1H, H(b)-C(5')); 3.88 (dd, J_1 = 3.11 Hz, J_2 = 7.31 Hz, 1H, H-C(4')); 4.03 (triplettoid, 1H, H-C(2')); 4.28 (dd, J_1 = 5.12 Hz, J_2 = 10.10 Hz, 1H, H-C(3')); 5.14 (triplettoid, 1H, HO-C(5')); 5.66 (d, J = 8.10 Hz, 1H, H-C(5)); 5.87 (d, J = 5.52 Hz, 1H, H-C(1')); 5.92 (d, J = 5.46 Hz, 1H, HO-C(3')); 7.85 (d, J = 8.10 Hz, 1H, H-C(6)); 11.38 (s, br, 1H, H-N(3)) ppm.

2'-Azido-5'-*O*-(4,4'-dimethoxytrityl)-2'-deoxyuridine (5)

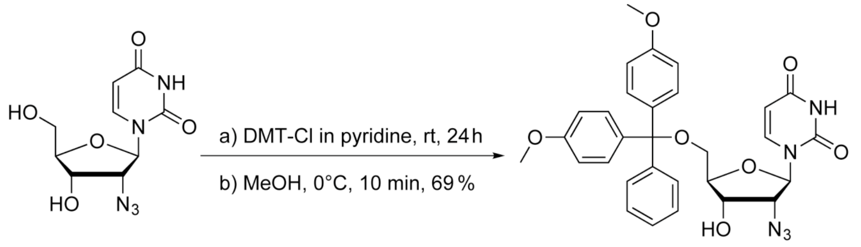

4

$C_9H_{11}N_5O_5$
[269.21]

5

$C_{30}H_{29}N_5O_7$
[571.58]

Compound **5** was prepared with slight modifications in analogy to the procedure described in reference 2: 2'-Azido-2'-deoxyuridine (**4**) was coevaporated twice with dry pyridine and dried overnight under vacuum. To a solution of compound **4** (238 mg, 0.88 mmol) in dry pyridine (7.2 mL) was added 4,4'-dimethoxytritylchloride (330 mg, 0.97 mmol). After 2 hours, another portion of 4,4'-dimethoxytritylchloride (150 mg, 0.44 mmol) were added and the reaction mixture was stirred at room temperature for 24 hours. The reaction was

quenched with MeOH (120 µL) at 0°C, stirred for 10 min and the solvents evaporated. The residue was dissolved in EtOAc (12 mL) and the organic layer washed with water (12 mL), saturated $NaHCO_3$ solution (12 mL) and water (12 mL). The combined organic layers were dried over Na_2SO_4 and evaporation led to the crude product, which was purified by column chromatography on SiO_2 (CH_2Cl_2/Et_3N, 99/1 v/v; packing the column; CH_2Cl_2/Et_3N, 99.5/0.5, v/v - CH_2Cl_2/MeOH/Et_3N, 98.5/1/0.5 v/v). Yield: 346 mg of **5** as white foam (69%). TLC (CH_2Cl_2/MeOH, 95/5): R_f = 0.54. ^{1}H NMR (300 MHz, $CDCl_3$): δ 3.49 (dd, J_1 = 2.54 Hz, J_2 = 11.24 Hz, 1H, H(a)-C(5')); 3.62 (dd, J_1 = 2.43 Hz, J_2= 11.22 Hz, 1H, H(b)-C(5')); 3.81 (s, 6H, 2 x O-CH_3); 4.04 (m, 1H, H-C(4')); 4.15 (dd, J_1 = 3.12 Hz, J_2 = 5.46, 1H, H-C(2')); 4.48 (triplettoid, 1H, H-C(3')); 5.38 (d, J = 8.16 Hz, 1H, H-C(5)); 5.99 (d, J = 3.06 Hz, 1H, H-C(1')), 6.86 (d, J = 8.85 Hz, 4H, H-C(ar)); 7.28-7.39 (m, 9H, H-C(ar)); 7.90 (d, J = 8.16 Hz, 1H, H-C(6)) ppm. ESI-MS (m/z): $[M+Na]^+$ calc for $C_{30}H_{29}N_5O_7$ 594.57; found 594.28.

2'-Azido-5'-*O*-(4,4'-dimethoxytrityl)-2'-deoxyuridine-3'-(2-chlorophenyl-phosphate) triethylammonium salt (1)

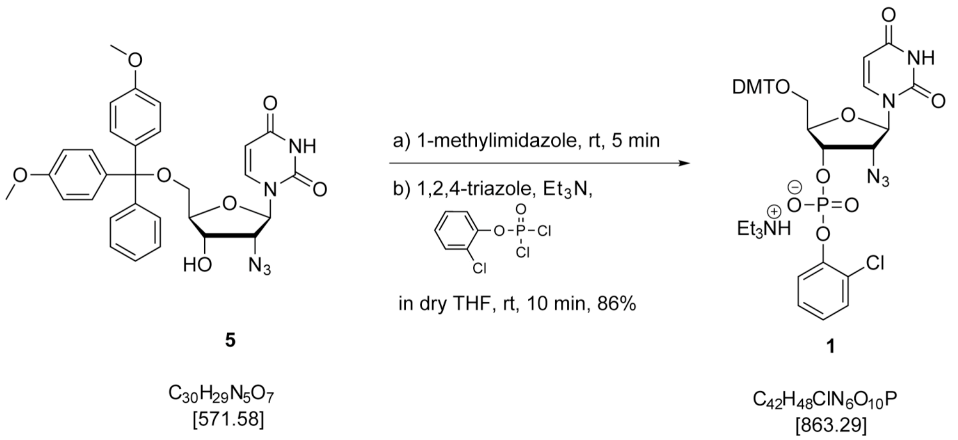

Compound **1** was prepared with slight modifications to a procedure described in reference 3 using 2'-azido-5'-*O*-(4,4'-dimethoxytrityl)-2'-deoxyuridine (**5**) instead of 5'-*O*-(4,4'-dimethoxytrityl)thymidine as starting material: 2-Chlorophenyl phosphorodichloridate (249 µL, 1.51 mmol) was added to a solution of 1,2,4-triazole (230 mg, 3.33 mmol) and triethylamine (422 µL, 3.03 mmol) in dry THF (14 mL) which led to a white precipitate. The obtained suspension was stirred at room temperature for 15 minutes. Then, a solution of compound **5** (346 mg, 0.61 mmol) in dry THF (4.6 mL) and 1-methylimidazole (193 µL, 2.42 mmol) were added and the reaction mixture was stirred for another 10 minutes at room temperature. As TLC (CH_2Cl_2/MeOH = 9/1) showed complete conversion the reaction was quenched with distilled water (154 µL) and triethylamine (844 µL, 6.10 mmol). Volatiles were evaporated and the residue was dissolved in CH_2Cl_2 and 10% aqueous $NaHCO_3$. The product was repeatedly extracted into CH_2Cl_2. The combined organic layers were washed with saturated NaCl solution, dried over Na_2SO_4 and the solvents were evaporated. The crude product was purified by column chromatography on SiO_2 (CH_2Cl_2/MeOH/Et_3N, 98/1/1, v/v for packing the column; CH_2Cl_2/MeOH/Et_3N, 98.5/1/0.5 – 93.5/6/0.5, v/v). Yield: 449 mg of **1** as white foam (86%). TLC (CH_2Cl_2/MeOH, 9/1): R_f = 0.49. ^{1}H NMR (300 MHz, $CDCl_3$): δ 1.20 (t, J = 7.28 Hz, 9H, $(CH_3CH_2)_3NH^+$); 2.86 (q, J = 7.26 Hz, 6H, $(CH_3CH_2)_3NH^+$); 3.46-3.51 (m, 1H, H(a)-C(5')); 3.53-3.57 (m, 1H, H(b)-C(5')); 3.76 (s, 3H, O-CH_3); 3.77 (s, 3H, O-CH_3); 4.33 (triplettoid, 1H, H-C(2')); 4.39 (m, 1H, H-C(4')); 5.15-5.21 (m, 2H, H-C(3'), H-C(5)); 6.02 (d, J = 4.17 Hz, 1H, H-C(1')); 6.79 (m, 4H, H-C(ar)); 6.94 (m, 1H, H-C(ar)); 7.09 (m, 1H, H-C(ar)); 7.21-7.34 (m, 10H, H-C(ar)); 7.58 (m, 1H, H-C(ar)); 7.77 (d, J = 8.18 Hz, 1H, H-C(6)) ppm. ^{13}C NMR (75 MHz, $CDCl_3$): δ 8.59 ($(CH_3CH_2)_3NH^+$); 45.82 ($(CH_3CH_2)_3NH^+$); 55.28 (2 x O-CH_3); 62.05 (C(5')); 65.62 (C(2'));

73.89 (C(3')); 82.55 (C(4')); 87.09; 87.31; 102.34 (C(5)); 113.33, 121.77, 123.88 (C(ar)); 125.24; 125.33; 127.14, 127.61, 128.02, 128.34 (C(ar)); 129.98; 130.23, 130.36 (C(ar)); 135.10; 135.25; 139.65 (C(6)); 144.30; 149.09; 149.17; 150.26; 158.67; 158.73; 162.99 ppm. ^{31}P NMR (121 MHz, $CDCl_3$): δ -5.71 ppm. ESI-MS (m/z): $[M+Et_3N]^+$ calc for $C_{42}H_{48}ClN_6O_{10}P$ 964.48; found 964.29.

^{1}H NMR (300 MHz, $CDCl_3$) of compound **1**:

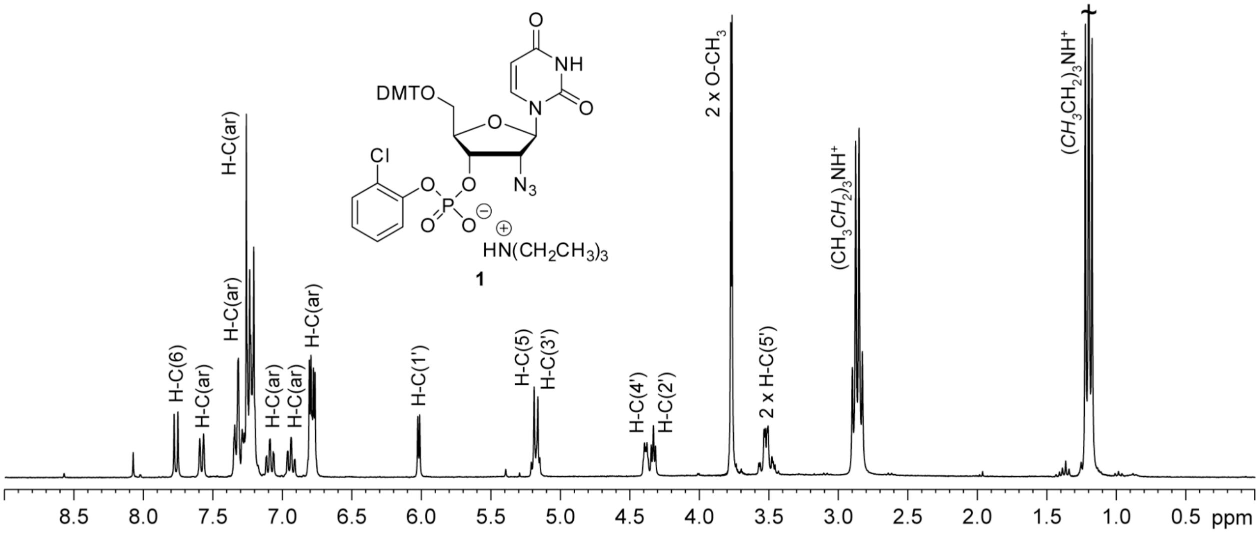

^{13}C NMR (75 MHz, $CDCl_3$) of compound **1**:

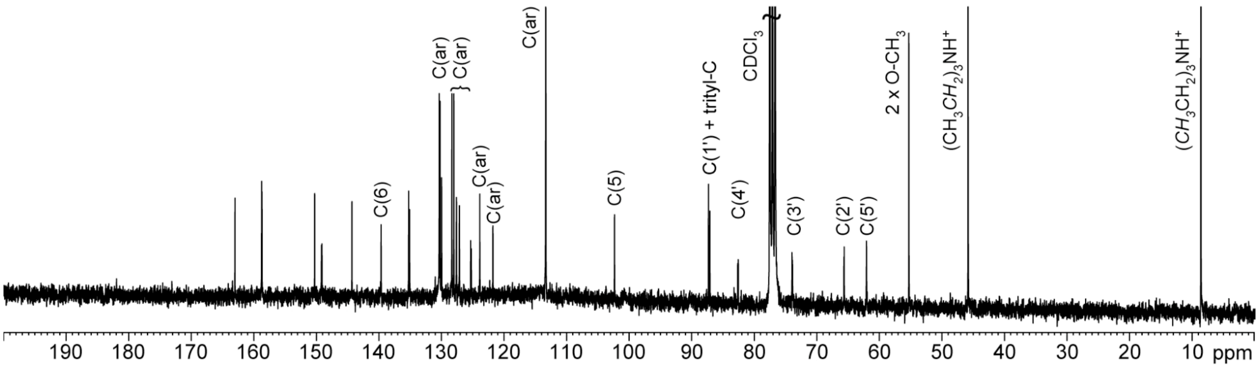

^{31}P NMR (121 MHz, $CDCl_3$) of compound **1**:

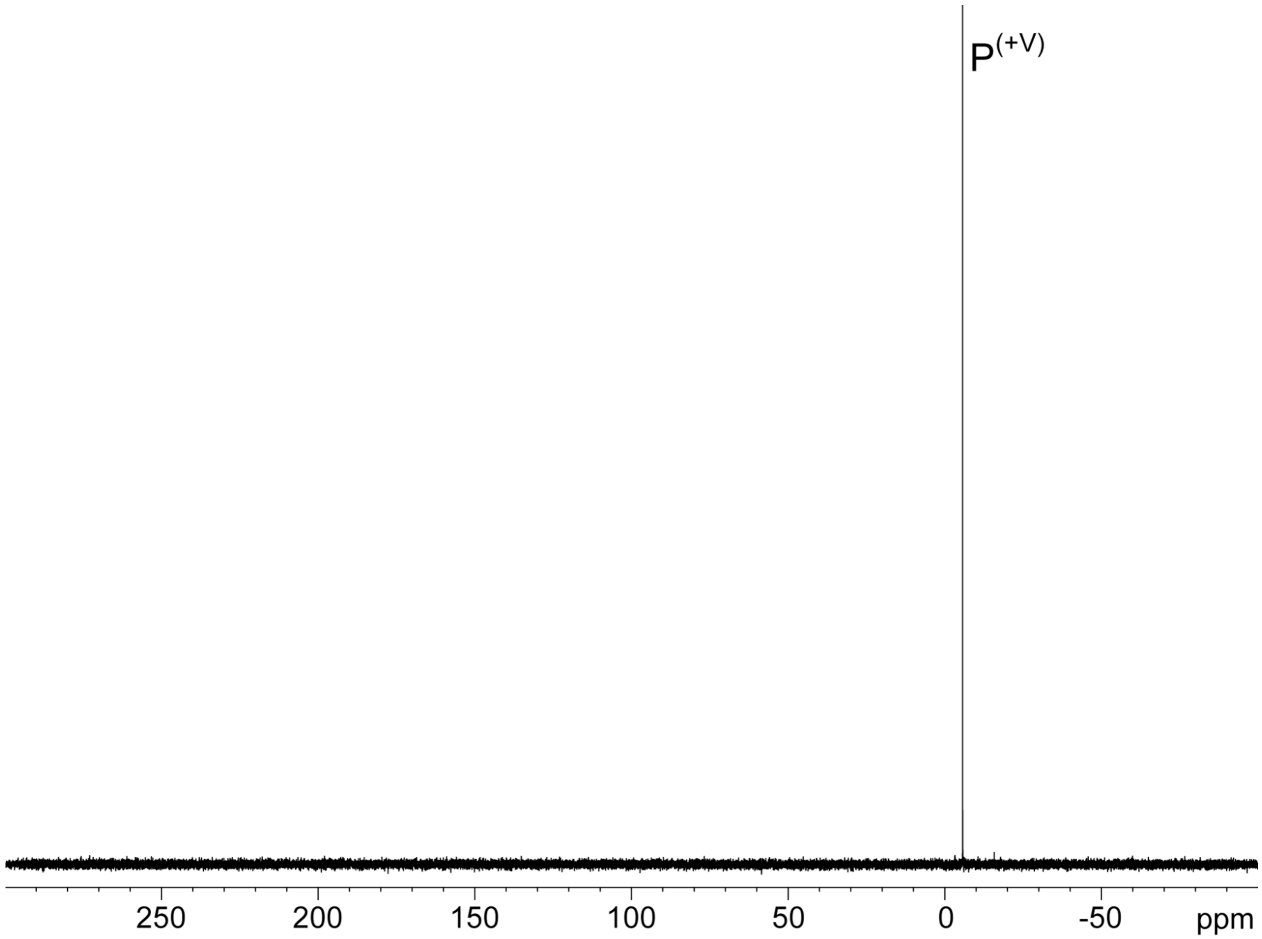

2. Synthesis of 2'-azido-2'-deoxyadenosine building block (2)

N[6]-Acetyl-2'-azido-2'-deoxy-3',5'-*O*-(1,1,3,3-tetraisopropyldisiloxane-1,3-diyl) adenosine (7)

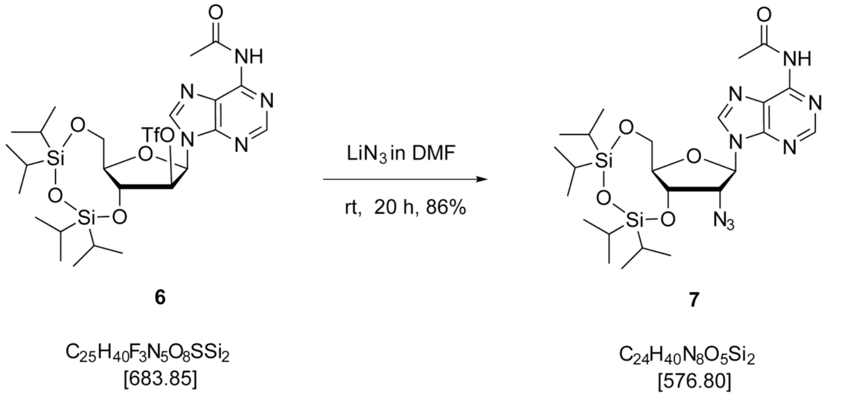

Starting compound **6** (1.81 g; 2.65 mmol) was prepared according to reference 4 and was dissolved in DMF (50 mL). Lithium azide (656 mg, 13.40 mmol) was added and the mixture was stirred for 20 hours at room temperature according to reference 5. After evaporation of the solvents the residue was dissolved in EtOAc and washed with 5% aqueous citric acid and saturated $NaHCO_3$ solution. The organic layer was dried over Na_2SO_4 and evaporated. The crude product was purified by column chromatography on SiO_2 (CH_2Cl_2/MeOH, 98/2, v/v). Yield: 1.31 g of **7** as slightly yellow foam (86%). TLC (CH_2Cl_2/MeOH, 94/6): R_f = 0.53. ^{1}H NMR (300 MHz, $CDCl_3$): δ 1.08-1.10 (m, 28H, 2 x ((CH_3)$_2$CH)$_2$Si); 2.63 (s, 3H, $COCH_3$); 4.12-4.26 (m, 3H, H(a)-C(5'), H(b)-C(5'), H-C(4')); 4.61 (d, *J* = 5.46 Hz, 1H, H-C(2')); 5.15 (dd, J_1 = 5.54 Hz, J_2 = 8.69 Hz, 1H, H-C(3')); 5.81 (s, 1H, H-C(1')); 8.17 (s, 1H, H-C(8)); 8.48 (s, br, 1H, H-N(6)); 8.65 (s, 1H, H-C(2)) ppm.

^{1}H NMR (300 MHz, $CDCl_3$) of compound **7**:

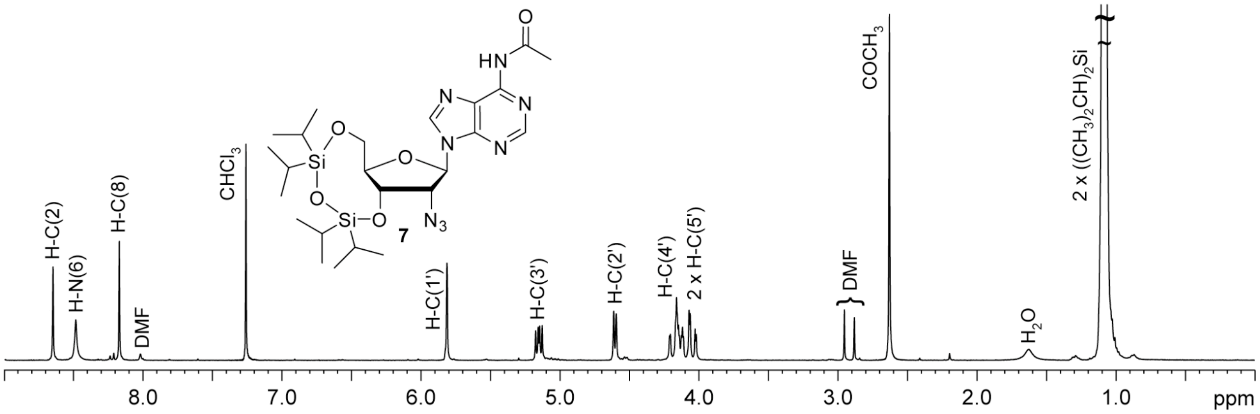

N^6-Acetyl-2'-azido-2'-deoxyadenosine (8)

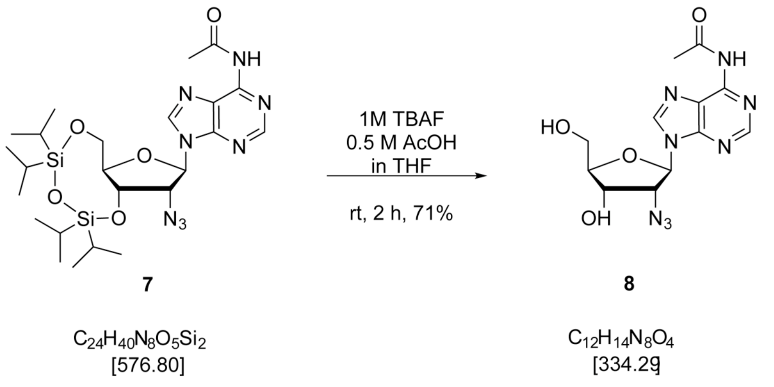

Compound **7** (1.31 g, 2.28 mmol) was treated with a mixture of 1.0 M tetrabutylammonium fluoride/0.5 M acetic acid in THF (8.9 mL). The solution was stirred at room temperature for 2 h and the reaction was monitored via TLC. Volatiles were evaporated and the residue was coevaporated three times with CH_2Cl_2 and dried under high vacuum. The crude product was purified by column chromatography on SiO_2 (CH_2Cl_2/MeOH, 100/0 – 95/5, v/v) which gave 665 mg of a white foam still containing equimolar amounts of tetrabutylammonium fluoride. Therefore the product was purified again by column chromatography on SiO_2 (CH_2Cl_2/MeOH, 98/2 – 95/5, v/v). Yield: 539 mg **8** as white foam (71%). TLC (CH_2Cl_2/MeOH, 9/1): R_f = 0.49. ^{1}H NMR (300 MHz, DMSO-d_6): δ 2.24 (s, 3H, $COCH_3$); 3.55-3.61 (m, 1H, H(a)-C(5')); 3.67-3.71 (m, 1H, H(b)-C(5')); 3.99 (m, 1H, H-C(4')); 4.57 (dd, J_1 = 5.04 Hz, J_2 = 9.87 Hz, 1H, H-C(3')); 4.69 (triplettoid, 1H, H-C(2')); 5.11 (triplettoid, 1H, HO-C(5')); 6.03 (d, J = 5.34 Hz, 1H, HO-C(3')); 6.12 (d, J = 5.58 Hz, 1H, H-C(1')); 8.65 (s, 1H, H-C(8) or H-C(2)); 8.68 (s, 1H, H-C(2) or H-C(8)); 10.68 (s, 1H, H-N(6)) ppm. ^{13}C NMR (75 MHz, DMSO-d_6): δ 24.29 ($COCH_3$); 60.72 (C(5')); 64.49 (C(2')); 70.88 (C(3')); 85.31 (C(1')); 85.92 (C(4')); 123.60; 142.44 (C(8)); 149.67; 151.47; 151.83 (C(2)); 168.85 ($COCH_3$) ppm. ESI-MS (m/z): $[M+H]^+$ calc for $C_{12}H_{14}N_8O_4$ 335.30; found 335.06.

^{1}H NMR (300 MHz, DMSO-d_6) of compound **8**:

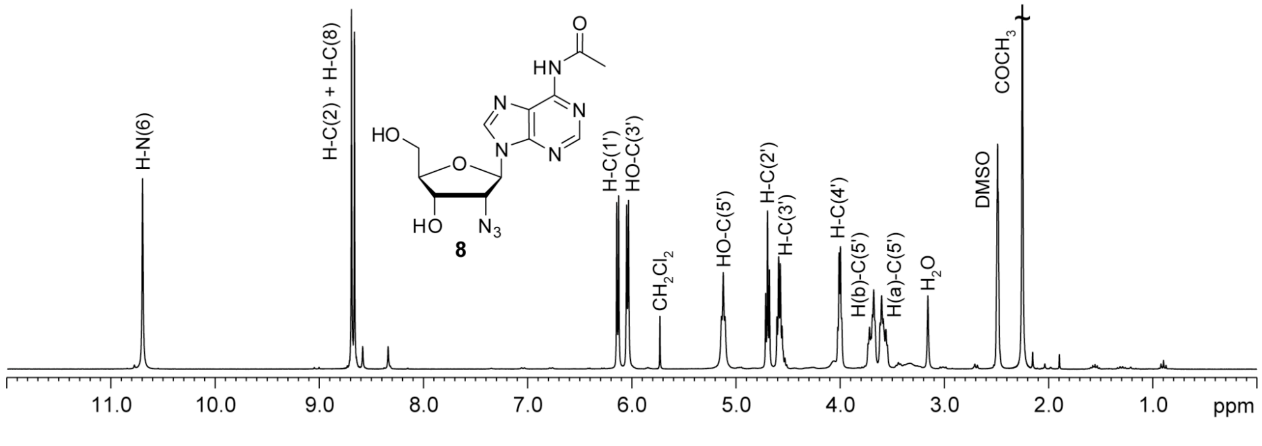

^{13}C NMR (75 MHz, DMSO-d_6) of compound **8**:

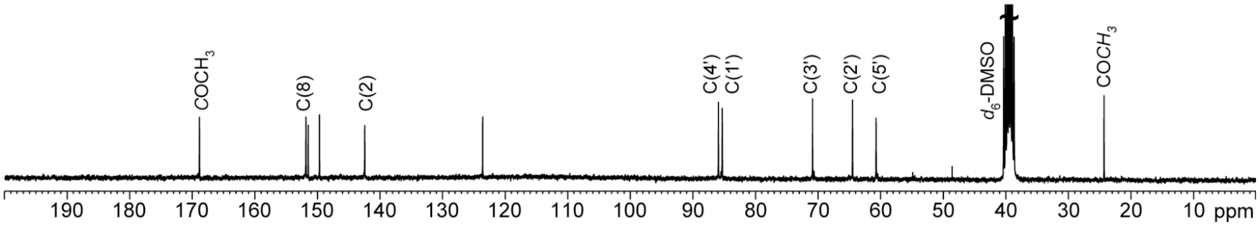

***N*⁶-Acetyl-2'-azido-5'-*O*-(4,4'-dimethoxytrityl)-2'-deoxyadenosine (9)**

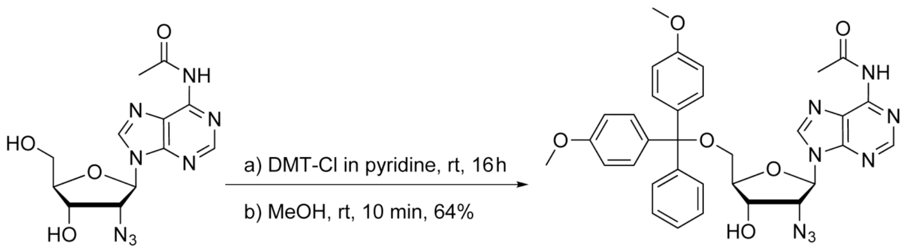

8 — $C_{12}H_{14}N_8O_4$ [334.29]

9 — $C_{33}H_{32}N_8O_6$ [636.66]

Compound **8** (351 mg, 1.05 mmol) was coevaporated twice with dry pyridine and dissolved in dry pyridine (4.3 mL). 4,4'-Dimethoxytritylchloride (534 mg, 1.57 mmol) was added in three portions over a period of 1 h and then stirred at room temperature for 16 h. After completion of the reaction methanol was added (335 µL) and the mixture was stirred for 10 min. Volatiles were evaporated and the residue was coevaporated twice with CH_2Cl_2. Then the residue was dissolved in CH_2Cl_2 and washed with 5% aqueous citric acid, water and saturated $NaHCO_3$ solution. The organic layer was dried over Na_2SO_4 and evaporated. The crude product was purified by column chromatography on SiO_2 (CH_2Cl_2/MeOH/Et_3N, 98/1/1, v/v for packing the column; CH_2Cl_2/MeOH/Et_3N, 98.5/1/0.5 – 97.5/2/0.5, v/v). Yield: 425 mg of **9** as white foam (64%). TLC (CH_2Cl_2/MeOH, 95/5): R_f = 0.47. ^1H NMR (300 MHz, $CDCl_3$): δ 2.56 (s, br, 1H, HO-(3')); 2.63 (s, 3H, $COCH_3$); 3.41 (dd, J_1 = 3.99 Hz, J_2 = 10.68 Hz, 1H, H(a)-C(5')); 3.52 (dd, J_1 = 3.87 Hz, J_2 = 10.71 Hz, 1H, H(b)-C(5')); 3.78 (s, 6H, 2 x O-CH_3); 4.22 (dd J = 4.02 Hz, 1H, H-C(4')); 4.65 (triplettoid, 1H, H-C(3')); 5.01 (triplettoid, 1H, H-C(2')); 6.08 (d, J = 5.28 Hz, 1H, H-C(1')); 6.80 (m, 4H, H-C(ar)); 7.21-7.30 (m, 7H, H-C(ar)); 7.37-7.40 (m, 2H, H-C(ar)); 8.12 (s, 1H, H-C(8)); 8.55 (s, br, 1H, H-N(6)); 8.60 (s, 1H, H-C(2)) ppm. ^{13}C NMR (75 MHz, $CDCl_3$): δ 25.77 (CO*CH₃*); 55.30 (2 x O-CH_3); 62.96 (C(5')); 65.62 (C(2')); 72.01 (C(3')); 84.07 (C(4')); 86.92 (C(1') or trityl-C); 87.26 (C(1') or trityl-C); 113.30 (C(ar)); 122.34; 127.11, 127.99, 128.17, 130.10 (C(ar)); 135.55; 135.58; 141.97 (C(8)); 144.43; 149.40; 150.92; 152.59 (C(2)); 158.72; 170.66 (*C*OCH_3) ppm. ESI-MS (m/z): $[M+H]^+$ calc for $C_{33}H_{32}N_8O_6$ 637.67; found 637.29.

^{1}H NMR (300 MHz, $CDCl_3$) of compound **9**:

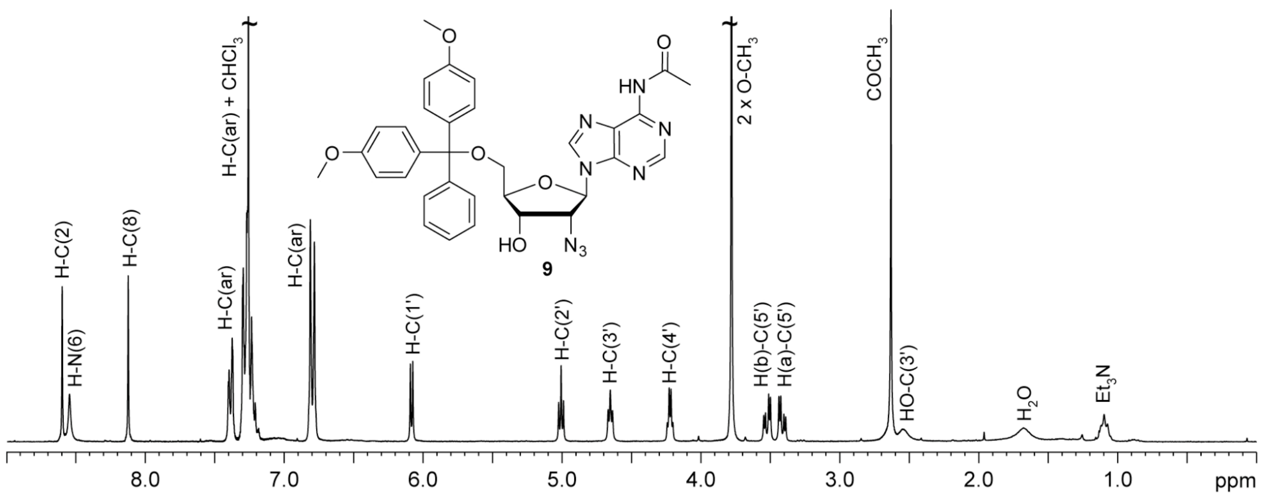

^{13}C NMR (75 MHz, $CDCl_3$) of compound **9**:

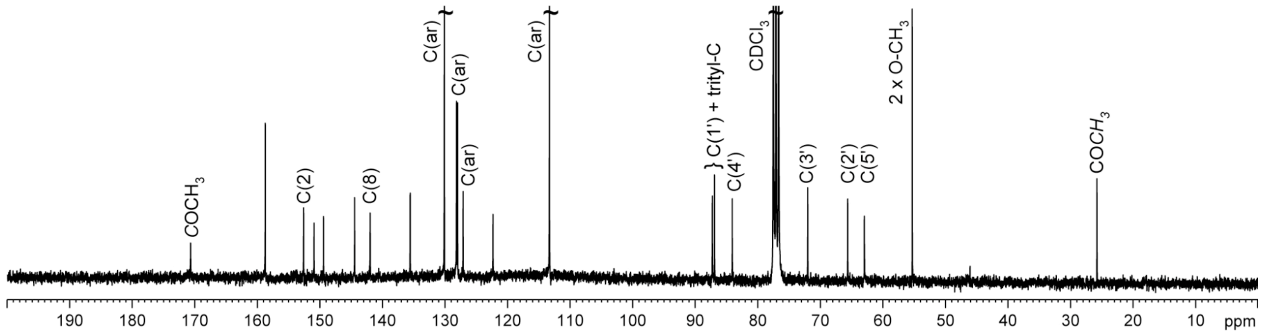

N^6-Acetyl-2'-azido-5'-*O*-(4,4'-dimethoxytrityl)-2'-deoxyadenosine-3'-(2-chlorophenylphosphate) triethylammonium salt (2)

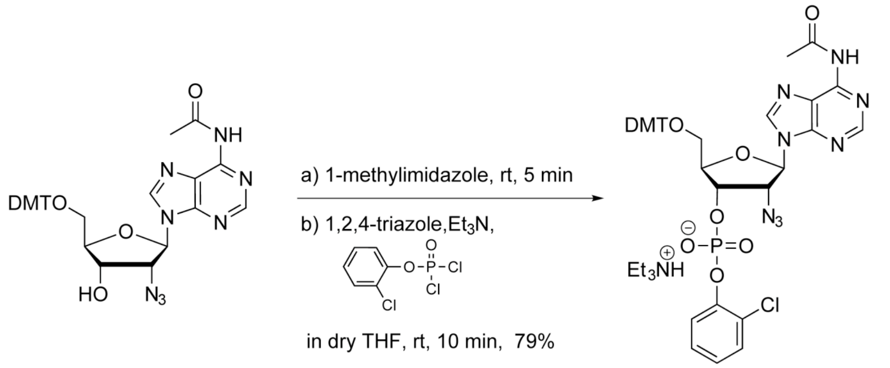

9 $C_{33}H_{32}N_8O_6$ [636.66]

2 $C_{45}H_{51}ClN_9O_9P$ [928.37]

The synthesis was performed as the synthesis of compound **1** according to reference 3. 2-Chlorophenyl phosphorodichloridate (308 µL, 1.87 mmol) was added to a solution of 1,2,4-triazole (284 mg, 4.11 mmol) and triethylamine (521 µL, 3.74 mmol) in dry THF (15 mL) which led to a white precipitate. The obtained suspension was stirred at room temperature for 15 minutes. Then a solution of compound **9** (425 mg, 0.67 mmol) in dry THF (8 mL) and 1-methylimidazole (238 µL, 2.99 mmol) were added and the reaction mixture was stirred for another 10 minutes at room temperature. As TLC (CH_2Cl_2/MeOH = 9/1) showed complete conversion the reaction was quenched with distilled water (191 µL) and triethylamine (1043 µL, 7.48 mmol). Volatiles were evaporated and the residue was dissolved in CH_2Cl_2 and 10% aqueous $NaHCO_3$. The product was repeatedly extracted into CH_2Cl_2. The combined organic layers were washed with saturated NaCl solution, dried over Na_2SO_4 and the solvent was evaporated. The crude product was purified by column chromatography on SiO_2 (CH_2Cl_2/MeOH/Et_3N, 98/1/1, v/v for packing the column; CH_2Cl_2/MeOH/Et_3N, 98.5/1/0.5 – 95.5/4/0.5, v/v). Yield: 492 mg of **2** as white foam (79%). TLC (CH_2Cl_2/MeOH, 9/1): R_f = 0.50. ^{1}H NMR (300 MHz, $CDCl_3$): δ 1.18 (t, *J* = 7.28 Hz, 9H, ($CH_3CH_2)_3NH^+$); 2.61 (s, 3H, $COCH_3$); 2.83 (q, *J* = 7.25 Hz, 6H, ($CH_3CH_2)_3NH^+$); 3.41 (dd, J_1 = 4.38 Hz, J_2= 10.71 Hz, 1H, H(a)-C(5')); 3.47 (dd, J_1 = 2.55 Hz, J_2= 10.62 Hz, 1H, H(b)-C(5')); 3.75 (s, 6H, 2 x O-CH_3); 4.54 (m, 1H, H-C(4')); 4.98 (triplettoid, 1H, H-C(2')); 5.23 (m, 1H, H-C(3')); 6.18 (d, *J* = 5.46 Hz, 1H, H-C(1')); 6.73 (m, 4H, H-C(ar)); 6.91 (m, 1H, H-C(ar)); 7.06 (m, 1H, H-C(ar)); 7.16-7.28 (m, 8H, H-C(ar)); 7.36 (m, 2H, H-C(ar)); 7.61 (m, 1H, H-C(ar)); 8.06 (s, 1H, H-C(8)); 8.56 (s, 1H, H-C(2)); 8.58 (s, br, 1H, H-N(6)) ppm. ^{13}C NMR (75 MHz, $CDCl_3$): δ 10.25 (($CH_3CH_2)_3NH^+$); 25.67 ($COCH_3$); 46.07 (($CH_3CH_2)_3NH^+$); 55.24 (2 x O-CH_3); 63.28 (C(5')); 64.71 (C(2')); 75.07 (C(3')); 83.63 (C(4')); 86.67 (C(1') or trityl-C); 86.79 (C(1') or trityl-C); 113.18 (C(ar)); 121.59 (C(ar)); 122.22; 123.77 (C(ar)); 125.07; 125.17; 126.94, 127.56, 127.86, 128.31 (C(ar)); 129.93; 130.19; 130.22; 135.57; 141.39 (C(8)); 144.47; 149.08; 149.18; 151.07; 152.49 (C(2)); 158.57; 170.40 ($COCH_3$) ppm. ^{31}P NMR (121 MHz, $CDCl_3$): δ -5.69 ppm. ESI-MS (m/z): $[M+Et_3N]^+$ calc for $C_{45}H_{51}ClN_9O_9P$ 1029.56; found 1029.27.

^{1}H NMR (300 MHz, $CDCl_3$) of compound **2**:

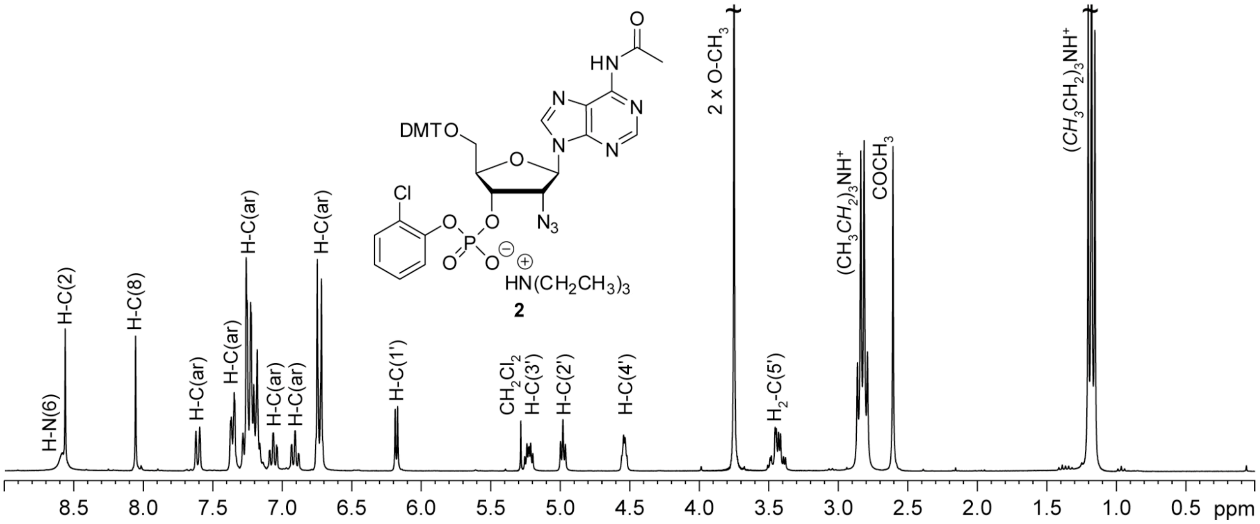

^{13}C NMR (75 MHz, $CDCl_3$) of compound **2**:

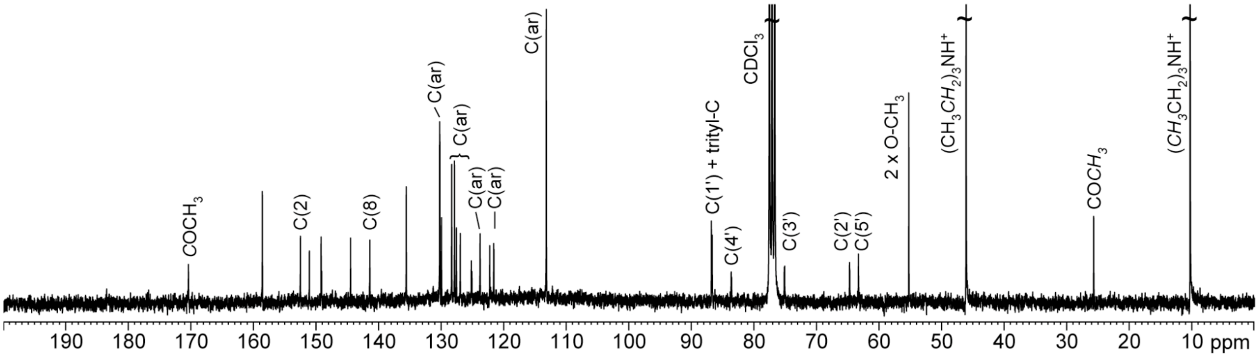

^{31}P NMR (121 MHz, $CDCl_3$) of compound **2**:

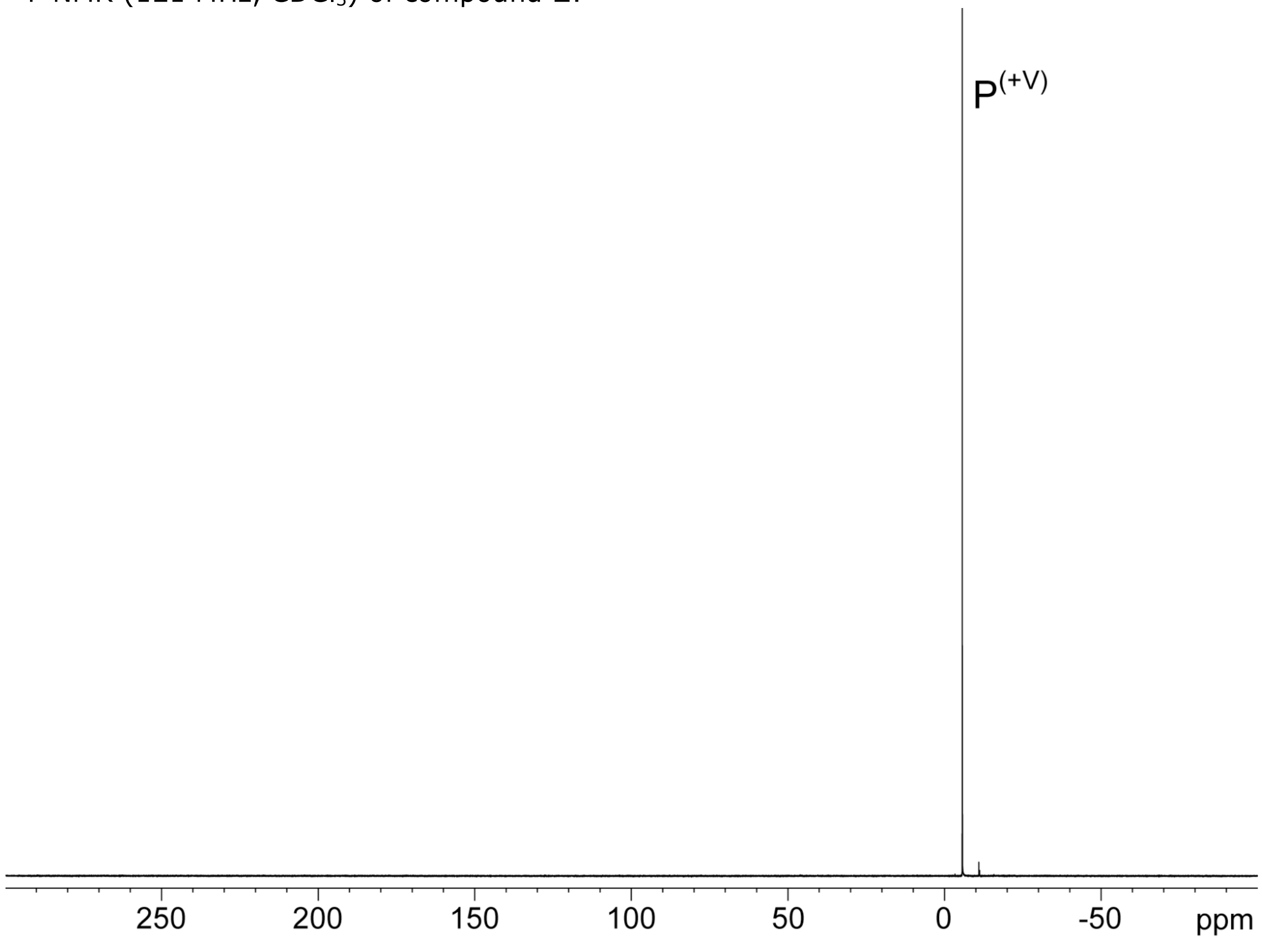

3. Solid-phase synthesis, deprotection and purification of 2'-azido-modified RNA

3.1. RNA solid-phase synthesis

Standard phosphoramidite chemistry was applied for RNA strand elongation up to the position of the 2'-azido nucleoside incorporation:
2'-*O*-TOM standard RNA nucleoside phosphoramidite building blocks were purchased from GlenResearch and ChemGenes and the corresponding polystyrene-supports from GE Healthcare (Custom Primer Support^{TM}, 80 µmol/g; *PS 200*). All oligonucleotides were synthesized on a Pharmacia Gene Assembler Special or Pharmacia Gene Assembler Plus following standard methods: detritylation (2.0 min): dichloroacetic acid/1,2-dichloroethane (4/96); coupling (3.0 min): phosphoramidites/acetonitrile (0.1 M x 120 µL) were activated by benzylthio-tetrazole/acetonitrile (0.3 M x 360 µL); capping (3 x 0.4 min): A: Ac_2O/*sym*-collidine/acetonitrile (20/30/50), B: 4-(dimethylamino)-pyridine/acetonitrile (0.5 M), A/B = 1/1; oxidation (1.0 min): I_2 (10 mM) in acetonitrile/*sym*-collidine/H_2O (10/1/5). Solutions of amidites, tetrazole solutions and acetonitrile were dried over activated molecular sieves (4 Å) overnight. All sequences were synthesized trityl-OFF.

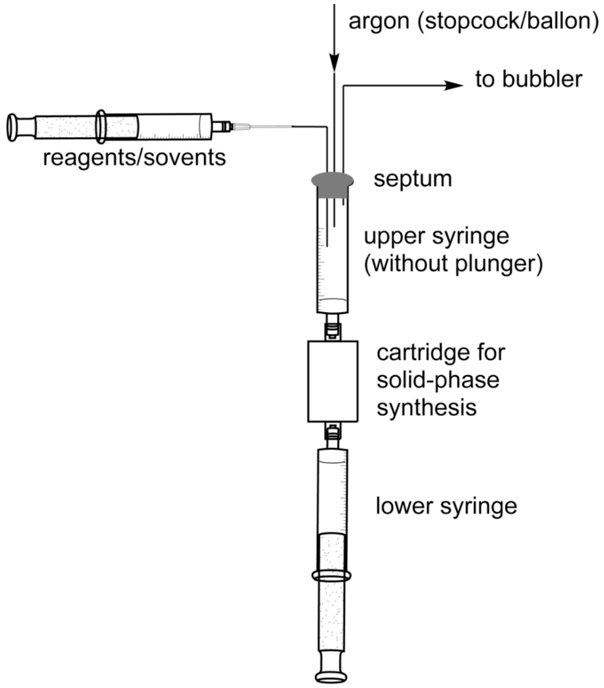

Supporting Figure 1. Schematics of the set-up for the manual coupling step of building blocks **1** and **2**.

Incorporation of 2'-azido nucleosides: Following phosphotriester chemistry, the respective building block, **1** or **2**, was manually coupled to the 5'-OH of the otherwise fully protected RNA chain attached to the solid support. The column was removed from the synthesizer and dried under high vacuum for 15 min. A solution of the appropriate building block **1** or **2** (0.3 M) in dry pyridine (250 µL), which had been dried over activated molecular sieves (4 Å) overnight, was activated by 1-(2-Mesitylenesulfonyl)-3-nitro-1*H*-1,2,4-triazole (MSNT; 0.9 M) for 10 min. Then, this reaction solution was applied on the column via an apparatus as shown in Supporting Figure 1. The coupling was performed under argon for one hour at room temperature and during this time the solution was moved through the column for several times. The solution turned brownish during the coupling time, was removed and

could be applied for another coupling step. The solid support in the column was washed with dry pyridine (1 mL) and dry acetonitrile (2 x 1 mL). Then, capping was performed manually too (solution A and B from standard synthesis, A/B = 1/1, 1 mL, 10 min at room temperature, the column was set between two syringes without applying argon). After this treatment the column was washed with dry acetonitrile (2 x 1 mL) and dried under high vacuum.
RNA strand elongation was continued by automated standard phosphoramidite chemistry starting with the detritylation step for removal of the 5'-protecting group of the manually coupled building block.

3.2. Deprotection of 2'-azido-modified RNA

First, cleavage of the 2-chlorophenyl group was performed according to reference 6 using a solution of *syn*-2-pyridinealdoxime/tetramethylguanidine (0.1 M for each) in dioxane/water (2:1, 1 mL). After 12 to 16 h at room temperature, the supernatant was removed from the solid support and the solid support was washed three times with dioxane/water (2/1, v/v). The solutions were combined and evaporated to dryness.
Both, the solid support and the residue from evaporation, were treated each with $MeNH_2$ in EtOH (33%, 0.65 mL) and $MeNH_2$ in water (40%, 0.65 mL) for 5 to 6 h at room temperature. The supernatant was removed from the solid support and the solid support was washed 3 x with ethanol/water (1/1, v/v). The supernatant and the washings were combined with the deprotection solution of the residue and the whole mixture was evaporated to dryness.
To remove the 2'-silyl protecting groups the resulting residue was treated with tetrabutylammonium fluoride trihydrate ($TBAF \cdot 3H_2O$) in THF (1 M, 1 mL) for at least 12 h at 37°C. The reaction was quenched by the addition of triethylammonium acetate (TEAA) (1 M, pH 7.4, 1 mL). The volume of the solution was reduced to 800 – 1000 µL and the solution was loaded on a size exclusion column (GE Healthcare, HiPrep™ 26/10 Desalting; 2.6 x 10 cm; Sephadex G25). The crude RNA was eluted with H_2O (controlled by UV detection at 260 nm and simultaneous detection of conductivity) and evaporated to dryness. Analysis of the crude RNA after deprotection was performed by anion-exchange chromatography on a Dionex DNAPac® PA-100 column (4 mm x 250 mm) at 80°C. Flow rate: 1 mL/min, eluant A: 25mM Tris·HCl (pH 8.0), 6 M urea; eluant B: 25 mM Tris·HCl (pH 8.0), 0.5 M $NaClO_4$, 6 M urea; gradient: 0-60 % B in A within 45 min or 0-40 % B in 30 min for short sequences up to 15 nucleotides, UV-detection at 260 nm.

3.3. Purification of 2'-azido-modified RNA

Crude RNA products were purified on a semipreparative Dionex DNAPac® PA-100 column (9 mm x 250 mm) at 80°C. Flow rate: 2 mL/min; gradient: Δ5-10% B in A within 20 min. Fractions containing RNA were loaded on a C18 SepPak Plus® cartridge (Waters/Millipore), washed with 0.1-0.15 M $(Et_3NH)HCO_3$, H_2O and eluted with H_2O/CH_3CN (1/1). RNA containing fractions were lyophilized. Analysis of the quality of purified RNA was performed by anion-exchange chromatography (same conditions as for crude RNA) and the molecular weight was confirmed by LC-ESI mass spectrometry. Yield determination was done by UV-photometrical analysis of oligonucleotide solutions.

3.4. Mass spectrometry of 2'-azido-modified RNA

All experiments were performed on a Finnigan LCQ Advantage MAX ion trap instrumentation connected to an Amersham Ettan micro LC system. RNA sequences were analyzed in the negative-ion mode with a potential of -4 kV applied to the spray needle. LC: Sample (250 pmol RNA dissolved in 30 µL of 20 mM EDTA solution; average injection volume: 30 µL); column (XTerra®MS, C18 2.5 µm; 1.0 x 50 mm) at 21°C; flow rate: 30 µL/min; eluant A: 8.6 mM TEA, 100 mM 1,1,1,3,3,3-hexafluoroisopropanol in H_2O (pH 8.0); eluant B: methanol; gradient: 0-100 % B in A within 30 min; UV-detection at 254 nm. Prior each injection, column equilibration was performed by eluting buffer A for 30 min at a flow rate of 30 µL/min.

4. Spectroscopic characterization of 2'-azido modified oligoribonucleotides

4.1. Thermal denaturation studies

Melting profiles (absorbance against temperature) were recorded at 250 nm, 260 nm, 270 nm and 280 nm on a *Varian* Cary 100 spectrophotometer equipped with a multiple cell holder and a Peltier temperature-control device. Data were collected after a complete cooling and heating cycle at a rate of 0.7°C/minute. Melting transitions were essentially the same with respect to the four different wavelengths. Melting data are fitted with polynom functions and T_m values are obtained from the first derivative.

Sample preparation:
Oligonucleotides (triethylammonium salts) were lyophilized to dryness, dissolved in melting buffer (10 mM Na_2HPO_4, 150 mM NaCl, pH 7.0) and subsequently degassed. A layer of silicon oil was placed on the surface of the solution.

Values of ΔH^0 and ΔS^0 for the bimolecular melting transition were derived from 1/T versus $\ln(c_T)$ plotting as described in literature[7]. Deviation for ΔH^0 and ΔS^0 arising from non-infinite cooperativity of two-state transitions and from the assumption of a temperature independent enthalpy are typically 10–15%. Additional deviation is introduced when free energies are extrapolated far away from the melting transitions; deviation for ΔG^0 are typically 3–5%.

4.2. CD spectroscopy

CD spectra were recorded on a *JASCO* J715 spectropolarimeter. Data were collected during 3 scans from 320 to 210 nm (100 nm/min).

Sample preparation:
Oligonucleotides (triethylammonium salts) were lyophilized to dryness, dissolved in melting buffer (10 mM Na_2HPO_4, 150 mM NaCl, pH 7.0) and subsequently degassed. All measurements are corrected to blank (melting buffer).

5. Staudinger ligation of 2'-azido-modified RNA for a molecular beacon application

5.1. Synthesis of the quencher derivative 10 (Disperse Red 1)

For the synthesis of quencher derivative **10**, references 8, 9 and 10 were taken into account.

4-*N*-Ethyl-*N*-(2-tosyloxyethyl)amino-4'-nitroazobenzene (16)

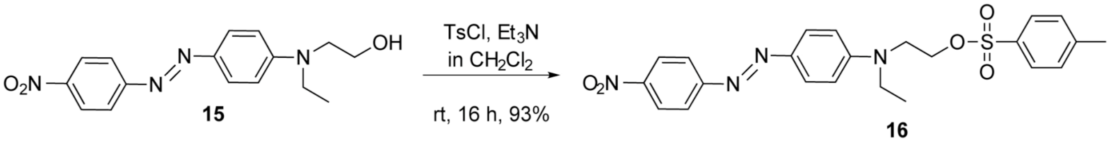

15 $C_{16}H_{18}N_4O_3$ [314.34]

16 $C_{23}H_{24}N_4O_5S$ [468.53]

To a solution of Disperse Red 1 (**15**) (185 mg, 0.59 mmol) in dry CH_2Cl_2 (2.0 mL) and Et_3N (167 µL, 1.2 mmol), *p*-toluenesulfonyl chloride (172 mg, 0.9 mmol) was added and the mixture was stirred for 16 hours at room temperature. Subsequently, the reaction mixture was diluted with CH_2Cl_2, washed twice with 1 M HCl solution, with H_2O and saturated NaCl solution. After drying of the organic phase over Na_2SO_4 the solvents were evaporated. The crude product was purified by column chromatography on SiO_2 (CH_2Cl_2). Yield: 256 mg of **16** as a red solid (93%). ^1H NMR (300 MHz, $CDCl_3$): δ 1.20 (t, 3H, J = 7.05 Hz, CH_2*CH_3*); 2.40 (s, 3H, ar-*CH_3*); 3.44 (q, 2H, J = 7.08 Hz, *CH_2*CH_3); 3.71 (t, 2H, J = 6.05 Hz, N-*CH_2*); 4.22 (t, 2H, J = 6.03 Hz, O-*CH_2*); 6.63 (d, 2H, J = 9.15 Hz, H-C(ar)); 7.27 (d, 2H, J = 8.61 Hz, H-C(ar));7.73 (d, 2H, J = 8.25 Hz, H-C(ar)); 7.84 (d, 2H, J = 9.12 Hz, H-C(ar)); 7.93 (d, 2H, J = 8.94 Hz, H-C(ar)); 8.33 (d, 2H, J = 8.91 Hz, H-C(ar)) ppm.

4-*N*-Ethyl-*N*-(2-azidoethyl)amino-4'-nitroazobenzene (17)

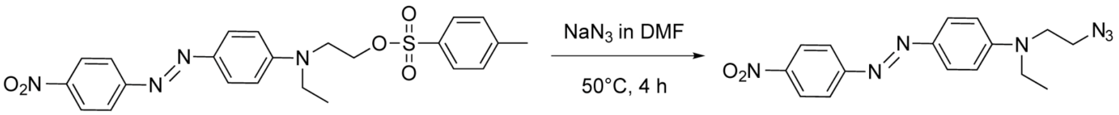

16 $C_{23}H_{24}N_4O_5S$ [468.53]

17 $C_{16}H_{17}N_7O_2$ [339.35]

To a solution of compound **16** (56 mg, 0.12 mmol) in dry DMF (2.5 mL) sodium azide (12 mg, 0.18 mmol) was added and the reaction mixture was stirred for 4 h at 50°C. After standing overnight at room temperature the solvent was evaporated and the residue was coevaporated twice with CH_2Cl_2 and dried under vacuum. The crude product **17** was used for the next step without further purification. TLC (EtOAc/hexane, 75/25): R_f = 0.36.

4-*N*-Ethyl-*N*-(2-aminoethyl)amino-4'-nitroazobenzene (18)

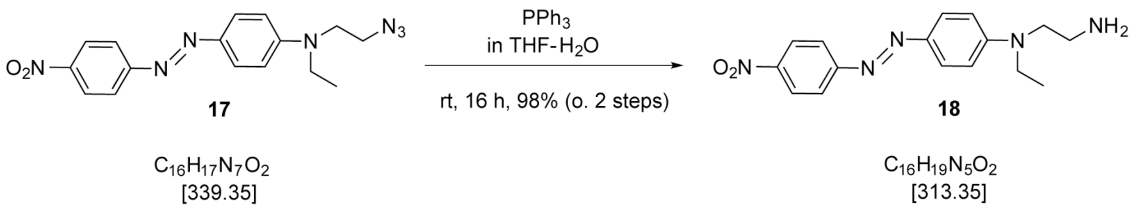

To a solution of crude compound **17** (prepared from 56 mg of **16**, 0.12 mmol) in THF (2.5 mL) were added triphenylphosphine (47 mg, 0.18 mmol) and H_2O (2.4 µL, 0.13 mmol). The reaction mixture was stirred at room temperature for 1 hour and an additional portion of water (2.4 µL, 0.13 mmol) was added and stirring continued overnight until complete conversion was obtained. The reaction mixture was evaporated to dryness and the crude product was purified by column chromatography on SiO_2 (EtOAc/MeOH, 9/1, v/v + 1% Et_3N). Yield: 37 mg of **18** as a red solid (98% over two steps). TLC (EtOAc/MeOH, 8/2 + 5% Et_3N): R_f = 0.26. ^1H NMR (300 MHz, $CDCl_3$): δ 1.24 (t, 3H, *J* = 7.04 Hz, CH_2*CH_3*); 3.00 (t, 2H, *J* = 6.89 Hz, N-*CH_2*); 3.48-3.57 (m, 4H, *CH_2*CH_3, N-C*H_2*); 6.78 (d, 2H, *J* = 9.21 Hz, H-C(ar)); 7.87-7.92 (m, 4H, H-C(ar)); 8.31 (d, 2H, *J* = 8.94 Hz, H-C(ar)) ppm. ^{13}C NMR (75 MHz, $CDCl_3$): δ 12.35 (CH_3); 39.92 (CH_2); 45.87 (CH_2); 53.51 (CH_2); 111.51, 122.67, 124.76, 126.38, 143.73, 147.59, 151.72, 157.07 (12 C(ar)) ppm. ESI-MS (m/z): $[M+H]^+$ calc for $C_{16}H_{19}N_5O_2$ 314.36; found 314.16.

^1H NMR (300 MHz, $CDCl_3$) of compound **18**:

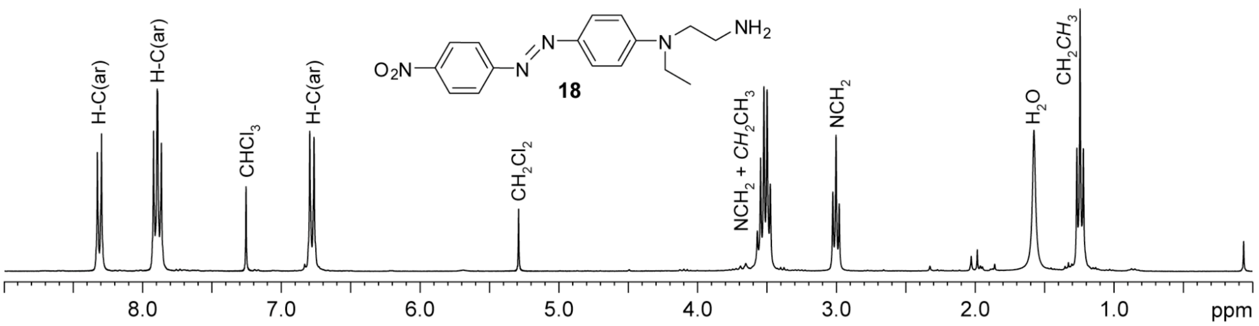

^{13}C NMR (75 MHz, $CDCl_3$) of compound **18**:

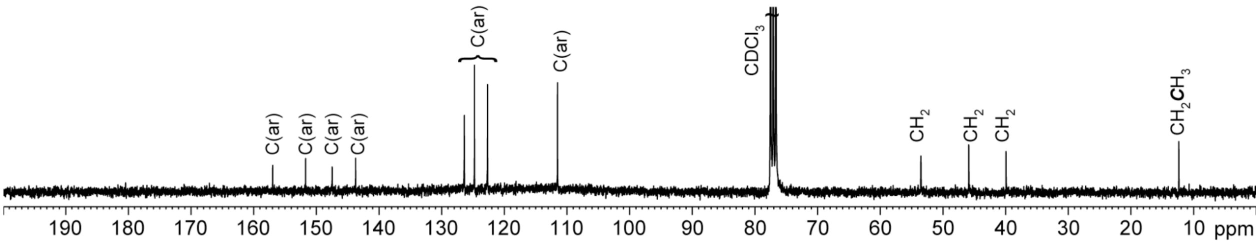

2-Diphenylphosphanyl-*N*-2-{*N*-ethyl-*N*-[4-(4-nitrophenylazo)phenyl]amino}ethyl-terephthalamic acid methyl ester (10)

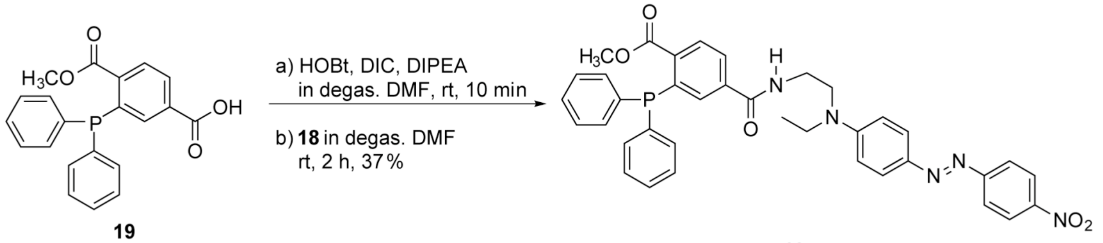

19

$C_{21}H_{17}O_4P$
[364.33]

10

$C_{37}H_{34}N_5O_5P$
[659.67]

To compound **19** (prepared according to reference 10) (70 mg, 0.19 mmol) and HOBt·H_2O (35 mg, 0.23 mmol) in dry, degassed DMF (1.0 mL), *N*,*N'*-diisopropylcarbodiimide (36 µL, 0.23 mmol) and *N*,*N*-diisopropyl-*N*-ethylamine (79 µL, 0.46 mmol) were added and the mixture was stirred for 10 min at room temperature for activation. Then, a solution of compound **18** (66 mg, 0.21 mmol) in dry, degassed DMF (1.5 mL) was added and the resulting reaction mixture was stirred for 2 h at room temperature. As TLC showed complete conversion, the DMF was evaporated and the residue dried under vacuum over night. The solid was dissolved in CH_2Cl_2 and washed with saturated $NaHCO_3$ solution and saturated NaCl solution. The organic layer was dried over Na_2SO_4 and the solvents were evaporated. The crude product was purified by column chromatography on SiO_2 (CH_2Cl_2/MeOH, 100/0 - 97/35, v/v). Yield: 47 mg of **10** as a red solid (37%). TLC (CH_2Cl_2/MeOH, 99/1): R_f = 0.77. ^{1}H NMR (300 MHz, $CDCl_3$): δ 1.18 (t, 3H, *J* = 6.99 Hz, CH_2CH_3); 3.42 (d, 2H, *J* = 6.90 Hz, CH_2CH_3); 3.58 (m, 4H, NCH_2CH_2N); 3.72 (s, 3H, O-CH_3); 6.29 (s, br, 1H, NH); 6.75 (d, 2H, *J* = 9.18 Hz, H-C(ar)(dye)); 7.26-7.31 (m, 11H, H-C(ar)); 7.76 (d, 1H, *J* = 7.68 Hz, H-C(ar)); 7.82 (d, 2H, *J* = 9.06 Hz, H-C(ar)(dye)); 7.87 (d, 2H, *J* = 8.91 Hz, H-C(ar)(dye)); 8.06 (dd, 1H, J_1 = 3.50 Hz, J_2 = 7.97 Hz, H-C(ar)); 8.25 (d, 2H, *J* = 8.91 Hz, H-C(ar)(dye)) ppm. ^{13}C NMR (75 MHz, $CDCl_3$): δ 12.33 (CH_3); 38.09 (CH_2); 45.79 (CH_2); 49.22 (O-CH_3) 52.31 (CH_2); 111.50, 122.70, 124.71, 126.34 (8C(ar)(dye)); 126.87, 128.68, 128.78, 129.05, 131.00, 132.37, 133.80, 134.08, 136.96, 137.12, 137.26, 141.57, 141.96 (18C(ar)); 143.85, 147.37, 151.28, 156.76 (4C(ar)(dye)); 166.67 (CO); 167.05 (CO) ppm. ^{31}P NMR (121 MHz, $CDCl_3$): δ -3.10 ppm. ESI-MS (m/z): $[M+H]^+$ calc for $C_{37}H_{34}N_5O_5P$ 660.68; found 660.30.

^{1}H NMR (300 MHz, $CDCl_3$) of compound **10**:

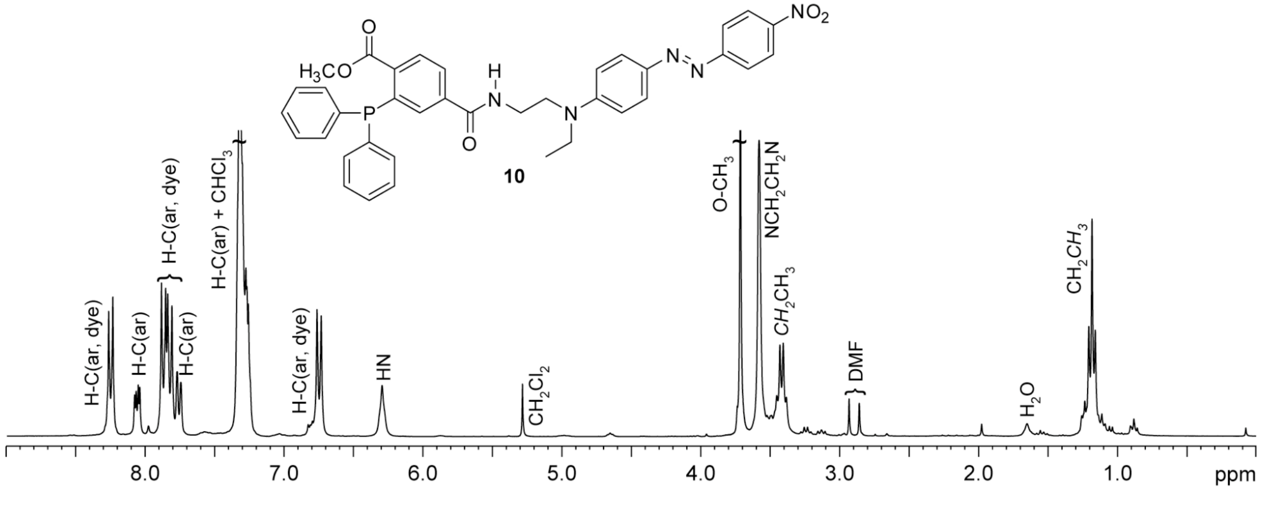

^{13}C NMR (75 MHz, $CDCl_3$) of compound **10**:

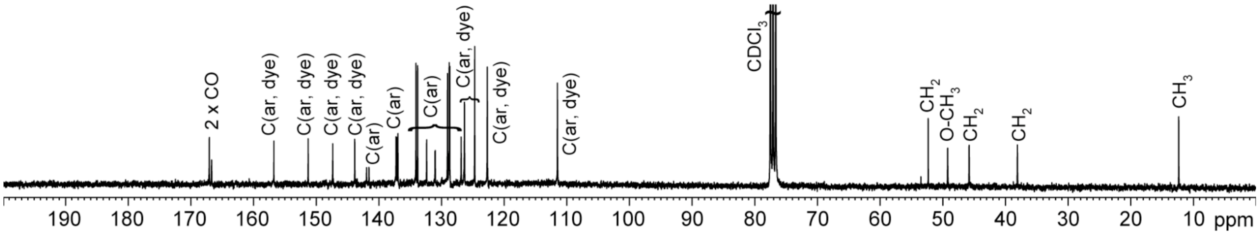

^{31}P NMR (121 MHz, $CDCl_3$) of compound **10**:

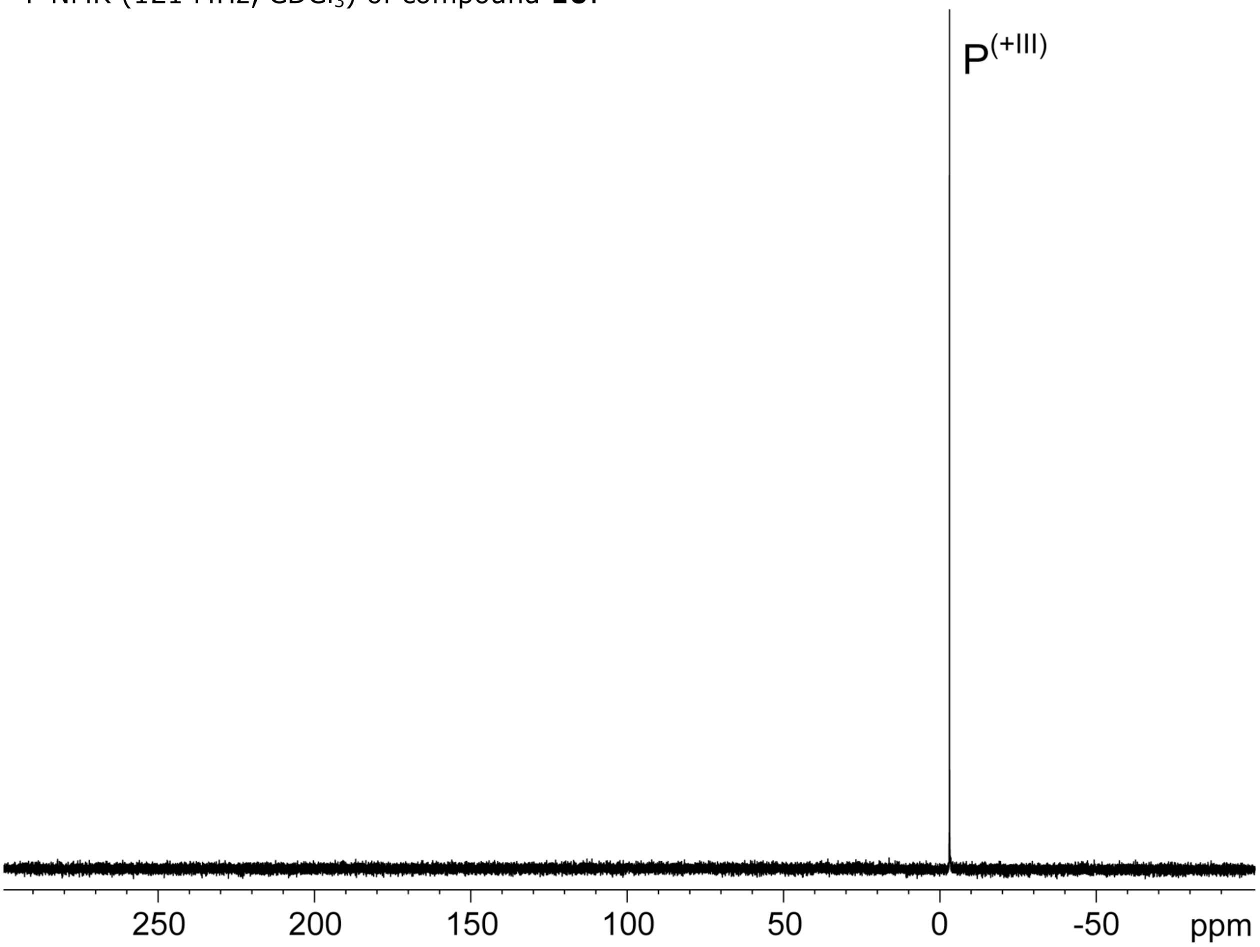

5.2. Staudinger ligation of derivative 10 to 2'-azido modified RNA

The Staudinger ligation of the 2'-azido modified RNA **11** and phosphine derivative **10** was performed in analogy to published procedures for DNA and proteins (references 11, 12, 13).

Typical ligation conditions: A solution of RNA **11** (3.75 nmol) in H_2O (25 µL; c_{RNA} = 150 µM) was mixed with DMF (56 µL) and a solution of phosphine derivative **10** in DMF (19 µL, $c_{quencher}$ = 20 mM) obtaining a final concentration of 37.5 µM RNA and 3.75 mM phosphine derivative (100 fold excess) in DMF/water (3/1, v/v). The mixture was incubated at 60°C for 21 h. Then the solvents were evaporated to dryness and the residue was dissolved in 2 mL of water and CH_2Cl_2. The aqueous layer was extracted three times with CH_2Cl_2 to remove the excess of phosphine derivative **10** and the combined organic layers were extracted with water. The combined aqueous layers were evaporated to dryness and the crude product was redissolved in 1.0 mL of water.
Analysis of the crude product was performed by anion-exchange chromatography on a Dionex DNAPac® PA-100 column (4 mm x 250 mm) at 80°C. Flow rate: 1 mL/min, eluant A: 25 mM Tris·HCl (pH 8.0), 6 M urea; eluant B: 25 mM Tris·HCl (pH 8.0), 0.5 M $NaClO_4$, 6 M urea; gradient: 0-60 % B in A within 45 min, UV-detection at 260 nm and additional detection at 490 nm and 502 nm (for the dyes). The HPLC trace showed complete conversion of the azide modified RNA **11** and the formation of three new products with different retention time (Supporting Figure 2).
The crude product was purified as described above (chapter 3.3) with separate collection of the obtained products. Analysis of the quality of the isolated products was performed by anion-exchange chromatography (same conditions as for crude product) and the assignment was done via the molecular weight (analyzed by LC-ESI mass spectrometry, as described in chapter 3.4; see also Supporting Figure 3).

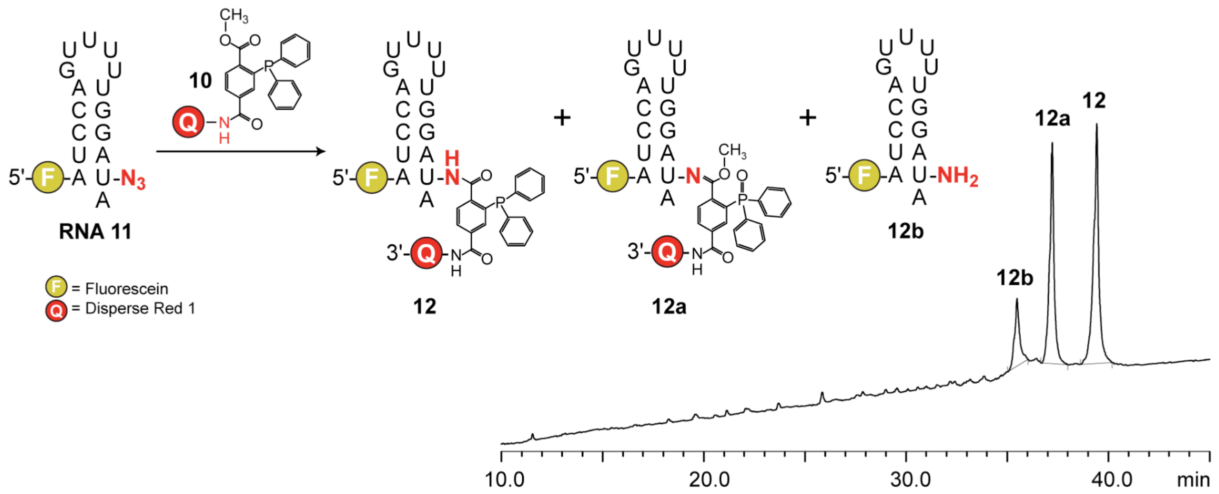

Supporting Figure 2. HPLC trace of the crude products of the Staudinger ligation. Staudinger ligation product **12** (amide-linked), Staudinger ligation product **12a** (imidate-linked)[14], and RNA byproduct **12b** (2'-NH_2 RNA from reduction of RNA **11**).

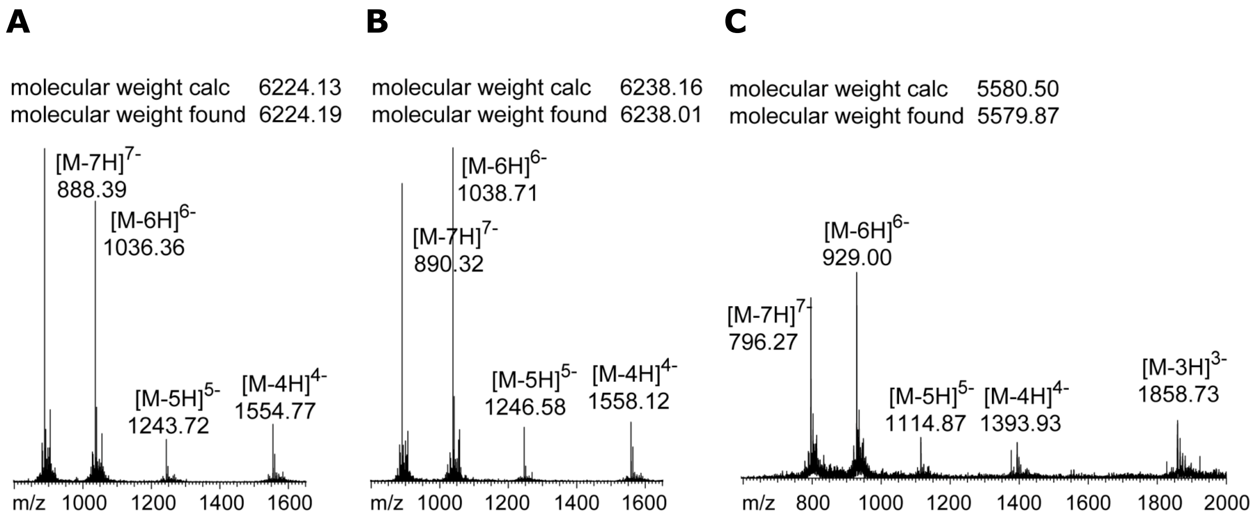

Supporting Figure 3. LC-ESI mass spectra of Staudinger ligation products. **A**) Staudinger ligation product **12** (amide-linked); **B**) Staudinger ligation product **12a** (imidate-linked); **C**) byproduct **12b** (2'-NH_2 RNA from reduction of RNA **11**).

6. Table of synthesized 2'-azido modified RNA sequences

No	Sequence[a]	isolated yield [nmol]	m.w. calc [amu]	m.w. obs[b] [amu]
1	5'-GGC **U**$^{2'N3}$UC AA-3'[c]	25	2534.6	2535.1
2	5'-GGC **U**$^{2'N3}$UC AA-3'	64	2534.6	2534.2
3	5'-GGC **U**$^{2'N3}$AC AA-3'	63	2557.6	2557.1
4	5'-GGC **U**$^{2'N3}$CC AA-3'	106	2533.6	2533.2
5	5'-GGC **U**$^{2'N3}$GC AA-3'	62	2573.6	2573.2
6	5'-GGU CG**A**$^{2'N3}$ CC-3'	196	2549.6	2549.2
7	5'-GG**U**$^{2'N3}$ CGA CC-3'	125	2549.6	2549.2
8	5'-GGC **U**$^{2'N3}$AG CC-3'	142	2549.6	2549.0
9	5'-CCA GGC C**U**$^{2'N3}$G G-3'	35[d]	3200.0	3200.2
10	5'-CC**A**$^{2'N3}$ GGC CUG G-3'	27[d]	3200.0	3200.4
11	5'-**U**$^{2'N3}$CG AAG UUA UCC-3'	35	3781.3	3781.0
12	5'-GAA GGG CAA CC**U**$^{2'N3}$ UCG-3'	173	4839.0	4838.4
13	5'-GA**A**$^{2'N3}$ GGG CAA CCU UCG-3'	161	4839.0	4838.3
14	5'-AUC CAG UUU UUG GA**U**$^{2'N3}$ A-3'	89	5069.1	5068.6
15	5'-**F**AU CCA GUU UUU GGA **U**$^{2'N3}$A-3'[e]	251	5606.5	5605.8
16	5'-*C*GU *AC*G *C*GG **U**$^{2'N3}$U*A* *AC*U U*C*G *A*UU-3'[f]	51	6801.3	6801.6
17	5'-CGU ACG CGG **U**$^{2'N3}$UA ACU UCG AUU-3'	45	6675.0	6675.2
18	5'-CGU ACG CGG UUA ACU UCG A**U**$^{2'N3}$U-3'	183	6675.0	6675.0
19	5'-**U**$^{2'N3}$CG AAG UUA UCC GCG UAC GUG-3'	13	6714.1	6713.4
20	5'-GGU CUC UGC CAA UAA GAC A**U**$^{2'N3}$T-3'	406	6680.1	6680.0
21	5'-GGU CUC **U**$^{2'N3}$GC CAA UAA GAC ATT-3'	17	6678.1	6678.1
22	5'-GGU CUC UGC CAA **U**$^{2'N3}$AA GAC ATT-3'	128	6678.1	6677.8
23	5'-UGU CUU AU**U**$^{2'N3}$ GGC AGA GAC CTdG-3'	19	6697.1	6696.4
24	5'-UGU CUU A**U**$^{2'N3}$U GGC AGA GAC CTdG-3'	12	6697.1	6696.4
25	5'-UGU CU**U**$^{2'N3}$ AUU GGC AGA GAC CTdG-3'	10	6697.1	6696.5
26	5'-UGU C**U**$^{2'N3}$U AUU GGC AGA GAC CTdG-3'	99	6697.1	6696.8
27	5'-UGU CUU **A**$^{2'N3}$UU GGC AGA GAC CTdG-3'	121	6697.1	6696.8
28	5'-UGU CUU AUU GGC **A**$^{2'N3}$GA GAC CTdG-3'	50	6697.1	6696.4
29	5'-UGU CUU AUU GGC AG**A**$^{2'N3}$ GAC CTdG-3'	107	6697.1	6696.4
30	5'-UGU C**U**$^{2'N3}$U AU**U**$^{2'N3}$ GGC AGA GAC C)TdG-3'	12	6722.1	6721.1
31	5'-UGU CUU AU**U**$^{2'N3}$ GGC **A**$^{2'N3}$GA GAC C)TdG-3'	68	6722.1	6721.2
32	5'-CU**U**$^{2'N3}$ GCU GAA GUG CAC ACA GCA AG-3'	42	7394.5	7394.0
33	5'-UGC **U**$^{2'N3}$CC UAG UAC GAG AGG ACC GGA GUG-3'	76	8752.3	8751.5
34	5'-UGC UCC UAG **U**$^{2'N3}$AC GAG AGG ACC GGA GUG-3'	86	8752.3	8751.1

[a] **U**$^{2'N3}$ = 2'-azido-2'-deoxyuridine; **A**$^{2'N3}$ = 2'-azido-2'-deoxyadenosine
[b] LC-ESI MS
[c] first trial of incorporation without using the apparatus in Figure 1
[d] half synthesis scale
[e] **F** = 5'-Fluorescein-phosphoramidite (6-FAM)
[f] *A* = 2'-methoxy-2'-deoxyadenosine; *C* = 2'-methoxy-2'-deoxycytidine

7. Table of 2'-azido modified siRNA duplexes

Synonym	siRNA duplex[a]	m.w. calc [amu]	m.w. obs[b] [amu]
SIR unmod	G GUC UCU GCC AAU AAG ACA TT<br>dGT C CAG AGA CGG UUA UUC UGU	6653.1<br>6672.1	6652.6<br>6671.8
SIR Az-U20 s	G GUC UCU GCC AAU AAG ACA U*T<br>dGT C CAG AGA CGG UUA UUC UGU	6680.1<br>6672.1	6680.0<br>6671.8
SIR Az-U7 s	G GUC UCU* GCC AAU AAG ACA TT<br>dGT C CAG AGA CGG UUA UUC UGU	6678.1<br>6672.1	6678.1<br>6671.8
SIR Az-U13 s	G GUC UCU GCC AAU* AAG ACA TT<br>dGT C CAG AGA CGG UUA UUC UGU	6678.1<br>6672.1	6677.8<br>6671.8
SIR Az-U9 as	G GUC UCU GCC A AU AAG ACA TT<br>dGT C CAG AGA CGG U*UA UUC UGU	6653.1<br>6697.1	6652.6<br>6696.4
SIR Az-U8 as	G GUC UCU GCC AA U AAG ACA TT<br>dGT C CAG AGA CGG UU*A UUC UGU	6653.1<br>6697.1	6652.6<br>6696.4
SIR Az-U6 as	G GUC UCU GCC AAU A AG ACA TT<br>dGT C CAG AGA CGG UUA U*UC UGU	6653.1<br>6697.1	6652.6<br>6696.5
SIR Az-U5 as	G GUC UCU GCC AAU AA G ACA TT<br>dGT C CAG AGA CGG UUA UU*C UGU	6653.1<br>6697.1	6652.6<br>6696.8
SIR Az-A7 as	G GUC UCU GCC AAU AAG ACA TT<br>dGT C CAG AGA CGG UUA* UUC UGU	6653.1<br>6697.1	6652.6<br>6696.8
SIR Az-A15 as	G GUC U CU GCC AAU AAG ACA TT<br>dGT C CAG A*GA CGG UUA UUC UGU	6653.1<br>6697.1	6652.6<br>6696.4
SIR Az-A13 as	G GUC UCU GCC AAU AAG ACA TT<br>dGT C CAG AGA* CGG UUA UUC UGU	6653.1<br>6697.1	6652.6<br>6696.4
SIR Az-U5,U9 as	G GUC UCU GCC A AU AA G ACA TT<br>dGT C CAG AGA CGG U*UA UU*C UGU	6653.1<br>6722.1	6652.6<br>6721.5
SIR Az-U9,A13 as	G GUC UCU GCC A AU AAG ACA TT<br>dGT C CAG AGA* CGG U*UA UUC UGU	6653.1<br>6722.1	6652.6<br>6721.2
SIR random	U CUG GGU CUA AGC CAA ACA UT<br>dGU A GAC CCA GAU UCG GUU UGU	6674.0<br>6655.1	6674.4<br>6655.0

[a] *U** = 2'-azido-2'-deoxyuridine, *A** = 2'-azido-2'-deoxyadenosine; all siRNA duplexes depicted contain overhangs of 2'-deoxynucleosides; in each row: first lane = sense strand in 5' → 3' direction, second lane = antisense strand in 3' → 5' direction.

[b] LC-ESI MS

8. Thermal denaturation study of 2'-azido-modified siRNA duplexes

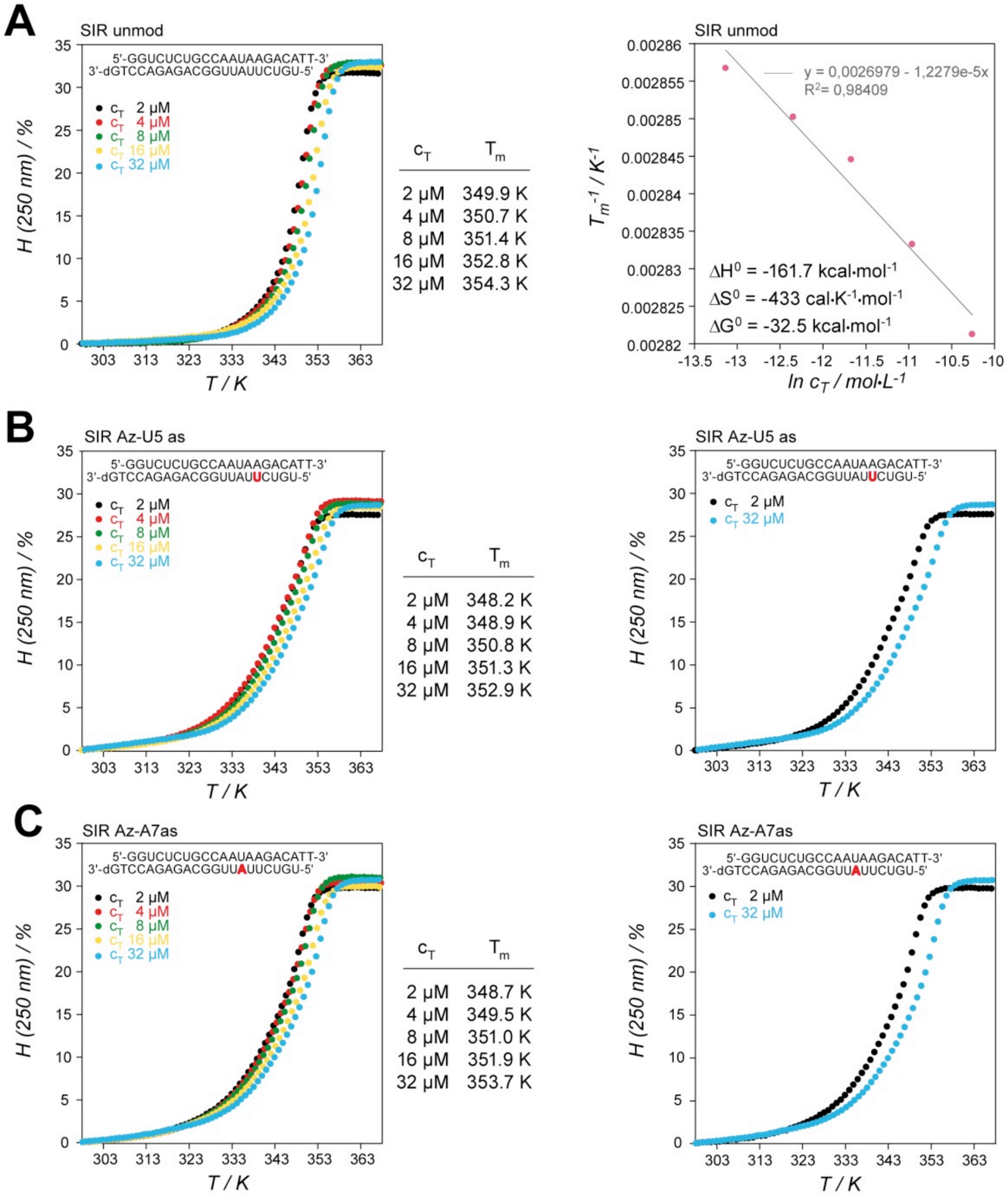

Supporting Figure 4. Comparison of thermal stabilities of unmodified and 2'-azido modified siRNA duplexes. **A**) Melting profiles (left) and thermodynamic analysis (right) of the unmodified siRNA duplex (SIR unmod); **B**) Melting profiles of the 2'-azido-2'-deoxyuridine modified siRNA duplex (SIR Az-U5 as) at five different concentrations (left) and at the lowest and highest concentrations for the sake of clarity (right); **C**) same as **B**) for the 2'-azido-2'-deoxyadenosine modified siRNA duplex (SIR Az-A7 as).

Melting data were fitted with polynom functions and T_m values were obtained from the first derivative. Conditions: c_T (total strand concentrations) as indicated, 10 mM Na_2HPO_4, 150 mM NaCl, pH 7.0.

In contrast to the unmodified siRNA, the melting profiles of the 2'-azido modified siRNA duplexes do not reflect a clear two-state melting behavior, and therefore no thermodynamic analysis was performed for the latter. To a first approximation, however, the melting profiles indicate that the 2'-azido modification may slightly decrease thermal stability (see also **Figure 3A** in the main text).

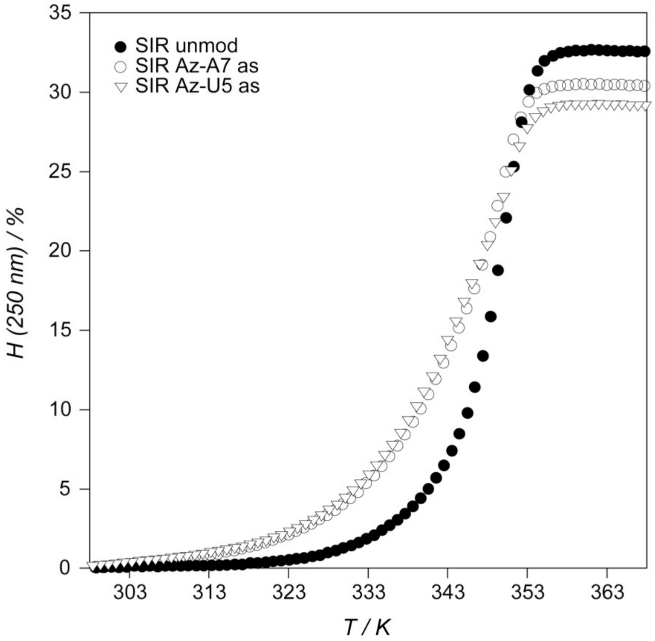

Supporting Figure 5. Overlay of UV melting profiles of the three different siRNA duplexes *SIRunmod*, *SIR Az-U5as* and *SIR Az-A7as*. Conditions: c_T = 4 μM, 10 mM Na_2HPO_4, 150 mM NaCl, pH 7.0.

9. RNA interference and Northern analysis

Delivery of siRNAs into cells and analysis of gene silencing were done essentially as described[15]. Lyophilized synthetic siRNA duplexes with the sense/antisense strands GGUCUCUGCCAAUAAGACATT/UGUCUUAUUGGCAGAGACCTdG targeted against the chicken *BASP1* mRNA sequence 5'-CAGGUCUCUGCCAAUAAGACA-3', or 2'-azido-modified derivatives thereof were dissolved in a buffer containing 100 mM potassium acetate, 30 mM Hepes-KOH (pH 7.4), and 2 mM magnesium acetate, yielding a 10 μM siRNA solution. The solution was heated at 90°C for 1 min, incubated at 37 °C for 1 h, and then stored at -20°C. For transfection of siRNA, 5 × 10^6 cells of the chicken fibroblast line DF-1 were pelleted at 50 × *g* for 5 min at room temperature, suspended in 100 μl of nucleofector solution V (Lonza/Amaxa), and mixed with 12 μL of siRNA solution containing 0.12 nmol (~1.5 μg) of duplex RNA. The mixture was subjected to electroporation (Lonza/Amaxa) using the nucleofector program U-20, and then immediately diluted with 0.5 mL of culture medium. Transfected cells were seeded onto 60-mm dishes containing 4 mL of culture medium and cultivated at 37°C. Medium was changed after one day, and total RNA was isolated after two days with the RiboPure Kit (Ambion). Briefly, cells were homogenized in a solution containing phenol and guanidine thiocycanate. After addition of bromochloropropane, RNA was recovered from the aqueous phase by binding to a glass-fiber filter and subsequent elution using a low-salt buffer. Northern analysis using 5 μg of total RNA and specific DNA probes for detection of *BASP1* or *GAPDH* mRNAs was performed as described previously[15,16]. Cell culture and DNA transfection of the retroviral RCAS-v-*myc* construct[17] into quail embryo fibroblasts (QEF) were done as described[15,16].

10. References

[1] a) S. F. Wnuk, S. M. Chowdhury, P. I. Garcia Jr., M. J. Robins. Stereodefined synthesis of O3′-labeled uracil nucleosides. 3′-[17O]-2′-Azido-2′-deoxyuridine 5′-diphosphate as a probe for the mechanism of inactivation of ribonucleotide reductases. *J. Org. Chem.* **2002**, *67*, 1816-1819; b) G. P. Kirschenheuter, Y. Zhai, W. A.Pieken. An improved synthesis of 2′-azido-2′-deoxyuridine. *Tetrahedron Lett.* **1994**, *35*, 8517-8520.

[2] M. A. Catry, A. Madder. Synthesis of functionalised nucleosides for incorporation into nucleic acid-based serine protease mimics. *Molecules* **2007**, *12*, 114-129.

[3] E. Belsito, A. Liguori, A. Napoli, C. Siciliano, G. Sindona. Straightforward synthesis of lipophilic thymidine glucopyranosyl monophosphates as models for a drug delivery system across cellular membranes. *Nucleosides, Nucleotides, Nucleic Acids* **1999**, *18*, 2565-2580.

[4] a) B. Puffer, H. Moroder, M. Aigner, R. Micura. 2'-Methylseleno-modified oligoribonucleotides for X-ray crystallography synthesized by the ACE RNA solid-phase approach. *Nucleic Acids Res.* **2009**, *37*, 7728-7740; b) C. Höbartner, R. Rieder, C. Kreutz, B. Puffer, K. Lang, A. Polonskaia, A. Serganov, R. Micura. Syntheses of RNAs with up to 100 nucleotides containing site-specific 2'-Se-methyl labels for use in X-ray crystallography *J. Am. Chem. Soc.* **2005**, *127*, 12035-12045.

[5] M. J. Robins, S. D. Hawrelak, A. E. Hernandez, S. F. Wnuk; Nucleic-Acid Related-Compounds .71. Efficient General-Synthesis of Purine (Amino, Azido, and Triflate)-Sugar Nucleosides; *Nucleosides & Nucleotides* **1992**, *11*, 821-834.

[6] R. Micura. Cyclic oligoribonucleotides (RNA) by solid-phase synthesis. *Chem. Eur. J.* **1999**, *5*, 2077-2082.

[7] L. A. Marky, K. J. Breslauer. Calculating Thermodynamic Data for Transitions of any Molecularity from Equilibrium Melting Curves. *Biopolymers*, **1987**, *26*, 1601-1620.

[8] J. Zhang, J. Drugeon, N. L’hermite. Synthesis of novel dendrimers incorporating a dye into the core. *Tetrahedron Lett.* **2001**, *42*, 3599-3601.

[9] D. Ghosh, L. Thander, S. K. Ghosh, S. K. Chattopadhyay. Sequential Aza-Claisen rearrangement and ring-closing metathesis as a route to 1-benzazepine derivatives. *Synlett* **2008**, *19*, 3011-3015.

[10] E. Saxon, C. R. Bertozzi. Cell surface engineering by a modified Staudinger reaction. *Science* **2000**, *287*, 2007-2010.

[11] K. L. Kiick, E. Saxon, D. A. Tirrell, C. R. Bertozzi. Incorporation of azides into recombinant proteins for chemoselective modification by the Staudinger ligation. *Proc. Natl. Acad. Sci. USA* **2002**, *99*, 19-24.

[12] A. Baccaro, S. H. Weisbrod, A. Marx. DNA conjugation by the Staudinger ligation: New thymidine analogues. *Synthesis* **2007**, *13*, 1949-1954.

[13] C. C.-Y. Wang, T. S. Seo, Z. Li, H. Ruparel, J. Ju. Site-specific fluorescent labeling of DNA using Staudinger ligation. *Bioconjugate Chem.* **2003**, *14,* 697-701.

[14] J. A. Restituyo, L. R. Comstock, S. G. Petersen, T. Stringfellow, S. R. Rajski, Conversion of Aryl Azides to O-Alkyl Imidates via Modified Staudinger Ligation. *Org. Lett.* **2003**, *5*, 4357-4360.

[15] M. Hartl, A. Nist, M. I. Khan, T. Valovka, K. Bister. Inhibition of Myc-induced cell transformation by brain acid-soluble protein 1 (BASP1). *Proc. Natl. Acad. Sci. USA* **2009**, *106*, 5604-5609.

[16] M. Hartl, F. Reiter, A. G. Bader, M. Castellazzi, K. Bister. *JAC*, a direct target of oncogenic transcription factor Jun, is involved in cell transformation and tumorigenesis. *Proc. Natl. Acad. Sci. USA* **2001**, *98*, 13601-13606.

[17] M. Hartl, A. M. Mitterstiller, T. Valovka, K. Breuker, B. Hobmayer, K. Bister. Stem cell-specific activation of an ancestral *myc* protooncogene with conserved basic functions in the early metazoan *Hydra*. *Proc. Natl. Acad. Sci. USA* **2010**, *107*, 4051-4056.
